# The Effect of Modifying the C9 Position of Fluorene with N-Donor Substituents on Selected Physicochemical Properties

**DOI:** 10.3390/molecules30091924

**Published:** 2025-04-25

**Authors:** Paweł Kalarus, Agata Szlapa-Kula, Michał Filapek, Sławomir Kula

**Affiliations:** Institute of Chemistry, Faculty of Science and Technology, University of Silesia, Szkolna 9 St., 40-007 Katowice, Poland; pawel.kalarus@us.edu.pl (P.K.); agata.szlapa-kula@us.edu.pl (A.S.-K.)

**Keywords:** fluorene derivatives, dibenzofulvene derivatives, Knoevenagel condensation, electrochromic materials, organic electronics, solar cells

## Abstract

Fluorene has been an extremely valued building block for many chemical compounds for a number of years. As a result, it is possible to design and obtain compounds with precisely defined physicochemical properties adapted to selected applications. An extremely interesting derivative of fluorene, which has been enjoying increasing interest in recent years, is dibenzofulvene (DBF) and its further structural modifications. So far, a number of dibenzofulvene derivatives have been described in the literature. Many of the presented DBFs are extremely structurally complex, which is why the influence of substituents on the physicochemical properties of the final compounds is not easy to determine unequivocally. Therefore, in this article, an attempt was made to explain the influence of N-donor substituents on selected physicochemical properties of dibenzofulvene derivatives (A-1–A-6). Moreover, these properties were compared to the results obtained for unsubstituted fluorene. The studies conducted showed that small modifications of the fluorene structure towards dibenzofulvene derivatives significantly change the absorption and emission properties of the final compounds. Importantly, the abovementioned structural modifications strongly affect the electrochemical properties, significantly reducing the energy gap and causing the oxidation potential to decrease to 0.18–0.42 V. Moreover, the process itself becomes fully reversible. The experimentally determined values coincide with those obtained theoretically via DFT calculations.

## 1. Introduction

**Figure 1:**
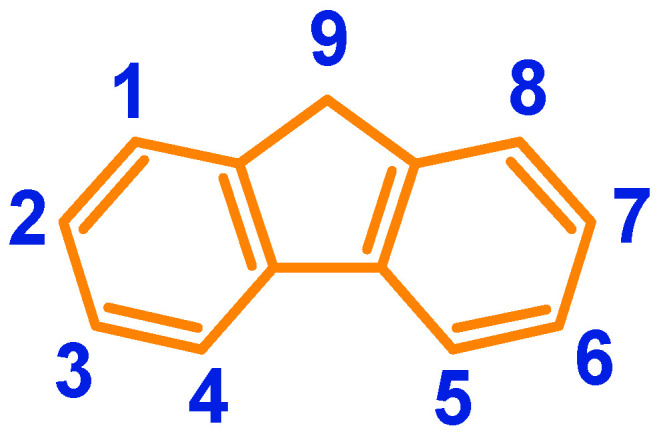
The most popular numbering of atomic positions in fluorene used in the synthesis of fluorene derivatives.

The search for chemical compounds with precisely defined physicochemical properties that allow their use in organic electronics and photovoltaics has been the focus of attention of scientists for a very long time. However, designing and obtaining a chemical compound that will exhibit specific physicochemical properties and simultaneously be processable and cheap to synthesize is not the easiest task. Fluorene has enjoyed great interest for many years, even decades. It is difficult to even list some of the research topics in which fluorene has been studied. However, what distinguishes the aforementioned compound from other aromatic derivatives is the fact that it is an extremely versatile building block. Fluorene can be successfully used to synthesize many extremely useful chemical compounds. Moreover, it can be modified using many chemical reactions, making it possible to obtain new chemical compounds with physicochemical properties tailored to needs. Fluorene reacts very well with chemicals in positions 2, 7, and 9 (Figure 1).

Positions 2 and 7 are often subjected to halogenation (bromination or iodination), and then to further reactions enabling the formation of the assumed derivatives [1,2,3]. In recent years, coupling reactions such as Suzuki, Sonogashira, and Buchwald–Hartwig have been very often used for this purpose [3,4,5,6,7,8,9]. Many reactions can also modify the 9-positions. In this case, a particularly interesting possibility is using the Knoevenagel condensation. This reaction allows the formation of a double bond at the 9-position of fluorene. Chemical compounds of this type are called dibenzofulvene derivatives (DBF) [10]. The second substrate necessary to carry out the aforementioned condensation is an aldehyde. Most often, aromatic or heteroaromatic aldehydes are used for this purpose, which allows the introduction of an additional substituent to the DBF structure that modifies the physicochemical properties of the final compound [10]. A wide selection of aromatic and heteroaromatic aldehydes available (even commercially) creates the possibility of obtaining an extremely wide group of dibenzofulvene derivatives. Moreover, the mentioned chemical compounds can be modified in positions 2 and 7, similar to fluorene [10]. Dibenzofulvene derivatives are becoming increasingly popular and not only because of their advantageous synthesis. Considering their attractive physicochemical properties, it is easy to indicate specific applications for which they have been studied in recent years. First of all, appropriately structurally modified dibenzofulvene derivatives are being studied for applications in solar cells [10,11,12,13,14,15,16,17,18,19,20,21]. In dye sensitized solar cells (DSSCs), dibenzofulvene derivatives are tested as organic dyes. Cells of this type using DBFs achieve an efficiency of up to 8% [10,11,12,13,21]. The second type of solar cells in which dibenzofulvene derivatives are studied are perovskite solar cells (PSCs) [10,14,15,16,17,18,19,20,21]. DBFs are tested as hole-transporting materials (HTM) in this type of cell. The considered PSC cells achieve efficiencies of up to 19–21% [10,14,15,16,17,18,19,20,21]. Interestingly, dibenzofulvene derivatives have been investigated as hole-transporting materials also in organic light-emitting diodes (OLEDs) [10,22]. Another important property of dibenzofulvene and its derivatives is the conjugated π-type bond system, which implies the ability of such compounds to undergo polymerization reactions under specific conditions. The polymerized DBF derivative is characterized by physicochemical properties, among which we can distinguish high thermal stability, high fluorescence efficiency, and appropriate charge transport [23,24,25,26,27,28,29,30,31]. These features are essential from the perspective of using dibenzofulvene derivatives in polymer light-emitting diodes (PLEDs) [10,23,25,32,33]. In addition, DBFs have been studied as highly efficient electrochromic materials capable of producing a wide range of colors [10,33].

Every year, we find more and more reports in the literature on dibenzofulvene derivatives studied for various applications. The compounds described in the latest publications are often very complex structurally, which makes it difficult to unequivocally assess the effect of individual substituents on the selected physicochemical properties. In this work, we focused on the impact of simple N-donor substituents on the physicochemical properties of dibenzofulvene derivatives. The compounds considered were compared in terms of optical and electrochemical properties. These properties are fundamental from the point of view of the applications for which DBFs are currently being studied. Moreover, the obtained research results were compared with the properties obtained for unsubstituted fluorene. Additionally, the experimental results obtained were compared using DFT calculations.

## 2. Result and Discussion

### 2.1. Synthesis

**Figure 2:**
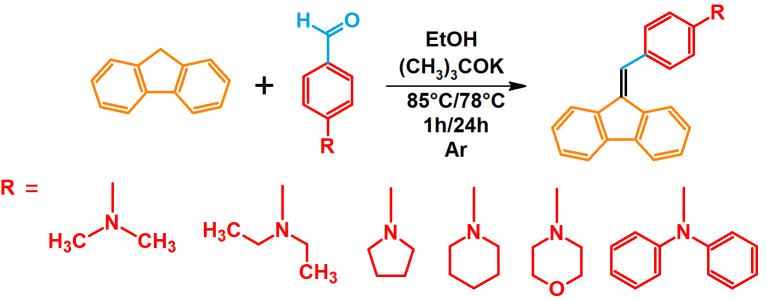
Synthesis of dibenzofulvene derivatives (A-1–A-6) by Knoevenagel condensation.

The studies began with the synthesis of the planned compounds. All dibenzofulvene derivatives (A-1–A-6) were obtained by Knoevenagel condensation according to an analogous procedure (Figure 2). The compounds’ synthesis began with preparing fluorene solutions in ethanol. Then, potassium tert-butoxide was added to them. The resulting mixtures were heated in an argon atmosphere for an hour. After this time, a three-fold excess of the appropriate aldehyde was added to each reaction mixture. Using an excess of aldehyde proved extremely beneficial because, in most experiments, the fluorene reacted completely. An excess of fluorene or using the exact proportions of fluorene and aldehyde resulted in difficult-to-separate post-reaction mixtures. After adding the necessary reagents, all reactions were heated at the boiling temperature of the reaction mixture, maintaining an argon atmosphere until the end of the experiments. The optimal time for all syntheses was 24 h. Shortening the reaction time resulted in a decrease in yield. On the other hand, the extension of the synthesis time did not positively affect the final results. The reaction products were isolated from the post-reaction mixtures and purified by extraction and column chromatography.

The structures of the obtained dibenzofulvene derivatives (A-1–A-6) were confirmed by NMR spectroscopic methods (^1^H and ^13^C). The necessary spectra are included in the Supporting Information (Section S3—Figures S1–S12). The structures, yields, and photographs of the obtained compounds are presented in Table 1.

### 2.2. Optical Properties

Studies of dibenzofulvene derivatives (A-1–A-6) began with recording the UV–Vis spectra. The compounds were studied in several solvents differing in their dielectric constant. This is because the polarity of solvents can affect the energy levels of the ground and/or excited states and consequently shift the emission/absorbance of the compound to a lower/higher wavelength [34]. In addition, the spectra of unsubstituted fluorene (A-0) were recorded. All samples were measured in solutions with a 5 × 10^−5^ mol/L concentration. The collected data are presented in Figure S18 and Table 2.

**Figure 3:**
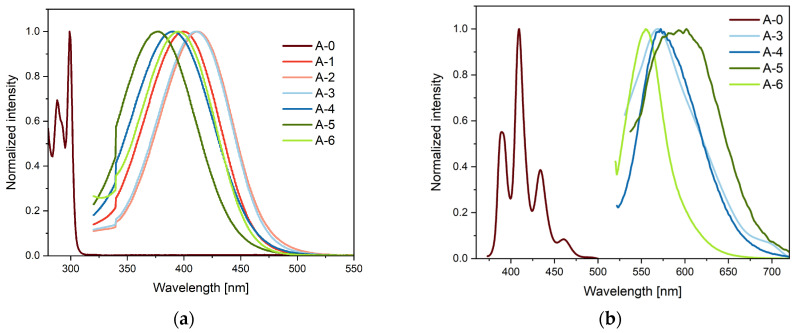
(**a**) Absorption spectrum of A-0–A-6 compounds recorded in ACN and (**b**) emission spectrum in the solid state.

The results obtained showed that compound A-0 has two distinct absorption maxima at λ = 289 and 301 nm (Figure S18). These sharp absorption bands correspond to the π–π* transitions for fluorene [11]. Despite using solvents of different polarity, the position of the maximum absorption band (λ_abs_) was essentially the same. This indicates the lack of solvatochromism for this compound. Moreover, it can be concluded that the optical gaps determined in different solvents for A-0 are the same because solvatochromism depends on the degree of charge separation in the molecule’s ground state. The results obtained for derivatives A-1–A-6 were analyzed successively. One broad absorption band in the range λ = 370–414 nm characterized them. The bands in the region of these wavelengths result from the intramolecular charge transfer (ICT) process. These molecules have similar absorption bands in the studied group. They also show similarity in this respect to structurally similar compounds described in the literature [11,34,35,36]. UV–VIS absorption studies of the entire group of derivatives showed that the change in solvent polarity had no significant effect. In turn, we see interesting relationships comparing the A-1–A-6 series with fluorene (A-0) (Figure 3a). Changing the structure of fluorene to dibenzofulvene and introducing an N-donor substituent to the structure of the compound results in a significant redshift of the absorption bands (around 76 nm—Figure S19 and Figure 3a). Moreover, this also changes the nature of the transitions of the last absorption band of these molecules. An important aspect of the research conducted was also the answer to the question of whether changing the nature of the N-donor substituent would affect the absorption bands. The presented derivatives show a wide range of λ_abs_ from 370–414 nm. As can be seen, the most red-shifted are A-2 and A-3. The most blue-shifted compound is A-5. It can be concluded that this is due to oxygen in the morpholine ring. This can be clearly seen when comparing A-5 with its analog A-4. The difference in the absorption band shift between these compounds is more than 10 nm. Similarly, when comparing A-1 with A-2, it is visible that the extension of the aliphatic chain causes a shift of the absorption maximum towards the red. Moreover, for compound A-0, emission maxima were determined in the 304–314 nm range. Similar values were obtained for fluorene derivatives [37]. All considered dibenzofulvene derivatives show weak fluorescence in solution relative to A-0. The emission quenching in compounds A-1–A-6 is probably due to the effective involvement of the ICT process [34]. Given the above, the emission of the tested compounds in the solid state was checked. It is clear from the obtained spectra that the modification of fluorene significantly affects the position of the emission bands (maximum shift by more than 100 nm—Figure 3b). The emission spectra in the solid state could not be recorded for compounds with an aliphatic chain.

### 2.3. DFT Calculations

**Figure 4:**
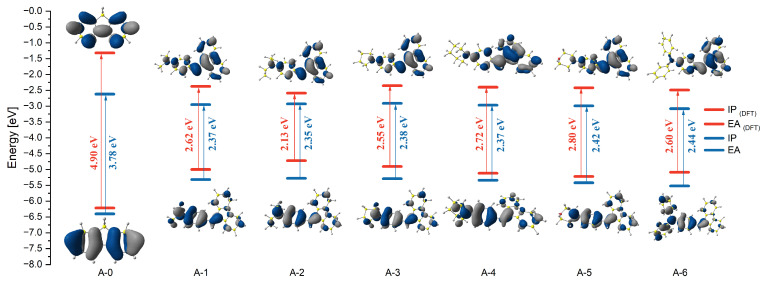
Comparison of the energy levels of molecular orbitals A-0–A-6 obtained theoretically and experimentally.

The density functional theory (DFT) calculations were carried out using the Gaussian16 software package [38] B3LYP/6-311+G** basis set [39,40,41,42,43,44,45,46,47] to understand the structural property of the compounds. On analyzing the optimized structures of compounds A-1–A-6, it could be observed that they exhibited non-planar configurations (Figure S20). The dibenzofulvene fragments and the N-donor substituent are bent/twisted relative to the plane, where the double bond and the phenyl ring are located (Figure S20). The dihedral angles between the substituent and the double bond were 124° (A-1–A-4) and 123° (A-5–A-6), respectively. The conformations adopted by the derivatives constitute a structural compromise that minimizes steric hindrance and the π-coupling path. However, the twisted structure may affect the photoluminescence in the discussed compounds [11]. The molecular orbital distributions of the HOMO and LUMO structures of all molecules are given in Figure S20. The HOMO electron clouds are mainly located on the N-donor substituent due to their ability to donate electrons. On the other hand, the LUMO electron clouds are dominated by the dibenzofulvene fragment (Figure 4). This fact is most visible in the A-6 molecule (Figure S20). Moreover, the ionization potential (IP) and electron affinity (EA) were calculated for the entire presented group (Table 3). The IP energy values in the A-1–A-6 group ranged from −4.72 to −5.22 eV. Comparing the obtained values with A-0 (fluorene), for which this value is −6.22 eV, it can be stated that the modification of fluorene to substituted dibenzofulvene causes significant changes. The same conclusion can be drawn for the EA energy values. For derivatives A-1–A-6, these values range from −2.35 eV to −2.59 eV, while for A-0, these values are −1.32 eV. This directly impacts the energy gap values ranging from 2.13 eV to 2.80 eV for A-1–A-6. For comparison, the energy gap of A-0 is 4.90 eV (Figure 4). Changing fluorene to substituted dibenzofulvene significantly reduces the energy gap, which is promising in terms of the application of these compounds.

In addition, UV–Vis absorption transitions were calculated using the TD-DFT method. These results suggest one major absorption band in the 350–500 nm range for A-1–A-6. The dominant calculated transition for the lowest energy absorption band for all derivatives is the S_0_ → S_1_ excitation. The significant increase in the oscillator strengths of this excitation can be attributed to the contribution of intramolecular charge transfer (ICT) transitions originating from charge delocalization from the substituent to the molecule’s core. Moreover, transitions that can be attributed to S_0_ → S_2_ excitations taking place within the core of the molecule are observed. The S_0_ → S_2_ transitions are characterized by very low oscillator strengths. UV–Vis transitions were simulated for the N-donor substituent group to make a more precise analysis of the absorption profile of the discussed compounds. Then, the absorption bands were assigned by comparing the calculated spectra of A-1–A-6 with those corresponding to their N-donor motifs (Figure S22). The bands appearing in the range of 200–350 nm show spectral features typical of the absorption of π–π* substituents. Compared to the free chromophores, only a small redshift is observed (Figure S22). Therefore, the absorption band in the range of 350–500 nm is caused by charge delocalization between the substituent and the molecule’s core. The simulated absorption spectra of the compounds showed good agreement with the experimental results (Figure S21).

### 2.4. Electrochemical and Spectroelectrochemical Properties

The electrochemical properties of the obtained compounds were investigated in MeCN solutions using cyclic voltammetry (CV). Using peak onset values determined from CV experiment allows for easy estimation of the molecules’ HOMO and LUMO energies (or, more precisely, the ionization potential (IP) and electron affinity (EA)) as well as the energy gap (Eg) (assuming the IP of ferrocene equals −5.1 eV) [48]. All the electrochemical parameters of the investigated compounds as well as the energy gaps obtained from CV (and DPV) measurements and DFT calculations are summarized in Table 3.

**Figure 5:**
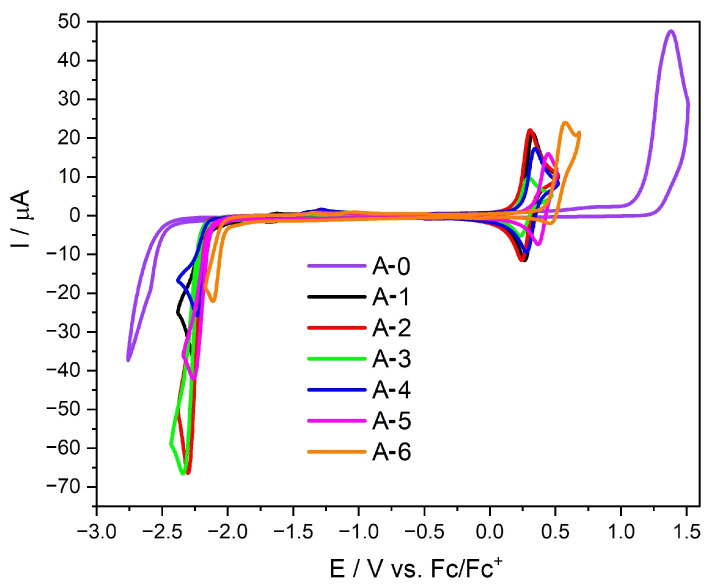
Cyclic voltammograms of the investigated compounds with sweep rate ν = 100 mV/s, 0.1 M Bu_4_NPF_6_ in MeCN.

The studied compounds belong to the fluorene derivatives with potential applications in modern technologies, primarily as hole-transporting materials (HTMs). Usually, fluorene derivatives are substituted in phenyl rings or di-substituted in positions 9 and 9’. This means that the linking carbon has sp^3^ hybridization, and thus, the physicochemical properties of this type of fluorene compound are similar to biphenyl derivatives. However, in the case of the derivatives described in this work, a molecular architecture was used that allows the 9th carbon to obtain the sp^2^ configuration, thus enabling the planarity of this molecule fragment. As expected, the abovementioned modifications cause significant facilitation of the electrode processes and, therefore, lower the energy band gaps (see Table 3 and Figure 5). Reduction of compounds A-1–A-6 occurs at lower values of external potential (by about 0.4 V) in relation to A-0. Moreover, what is particularly important is that oxidation occurs at a potential between 0.18 (for A-2) and 0.42 (for A-6), i.e., with values similar to those observed for structurally similar dibenzofulvene-triphenylamine derivatives [16,18]. It is also worth emphasizing that in each case, the oxidation process becomes thermodynamically reversible, proving the molecule’s high stability after p-doping—derivatives without stabilizing substituents show low stability after oxidation [10].

The energy gaps for the entire series of compounds have similar values, and one can also notice the regularity of the influence of amine substituents. Substituents that strongly affect the decrease in the E_ox_ value (i.e., increase the electron density) simultaneously make the reduction more difficult. This behavior can be seen best by comparing compounds A-2 and A-6. The first undergoes oxidation at the lowest potential (0.18 V), while it is reduced at −2.17 V. In turn, A-6 is the opposite, i.e., it is most difficult to oxidize (at 0.42 V), while the reduction occurs the easiest of all the investigated molecules (at −2.02 V). Moreover, since the change of the substituent affects both processes and, thus, indirectly, the energies of the HOMO and LUMO orbitals, it means that both orbitals (at least partially) are overlapping (see also Section 2.3 DFT calculations). Moreover, comparing the energy gaps determined by the electrochemical method with those calculated from the UV–Vis spectrum, it can be seen that the latter is overestimated for the entire series by about 0.3 V (see Table 3). This is a relatively small value (the higher this value would be, the greater the difficulty of charge flow within the molecule).

**Figure 6:**
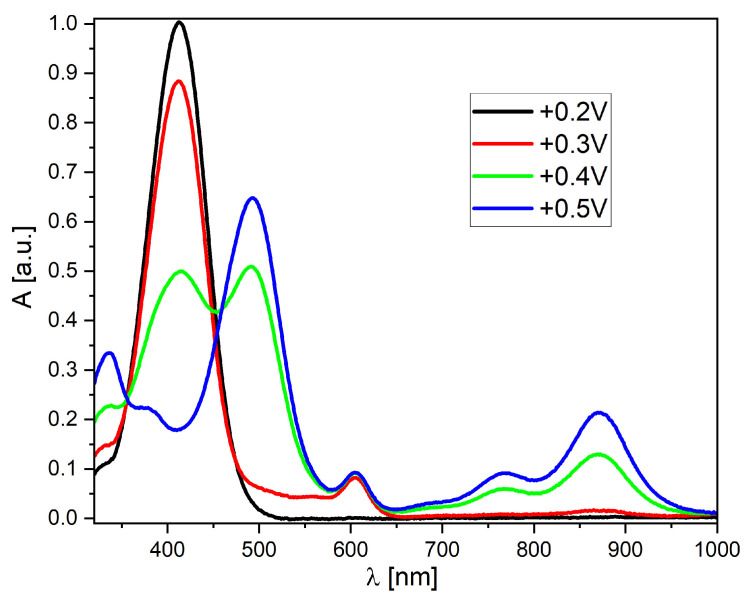
UV–Vis spectro-electrochemistry of the A-2 derivative in MeCN solution (c = 1 × 10^−5^ mol/L, as an inset on each graph, all potentials vs. Fc/Fc^+^ redox couple).

As mentioned earlier, the obtained derivatives show high stability of the oxidized forms. Therefore, a series of measurements using UV–Vis spectro-electrochemistry was performed (Figure 6 and Figure S23). Derivative A-2 was chosen as a representative example (Figure 6). This compound, in its neutral form, is characterized by one absorption band at the border of the visible range (peak maximum at 412 nm). This peak begins to disappear after exceeding the oxidation potential, while peaks appear and grow in the lower energy part of the spectrum. At a potential of +0.5 V, the highest value is obtained by the peak with a maximum at 492 nm. It is also worth noting the formation of a broad band covering the spectral region from 550 to 950 nm (with three local maxima). Such properties are similar to the properties of conducting polymers and result from the dispersion of positive charge within the conjugated system of double and single bonds. Therefore, this is a highly beneficial property from the point of view of further (potential) applications of the discussed group of compounds.

## 3. Experimental Data—Materials, Methods, and Other Research Results

All experimental results in the form of spectra, thermograms, graphs, and photos confirming the research described in the article are documented in the Supporting Information (SI). In addition, the materials, methods, and experimental procedures used in the research are described in detail there [49]. The following solvent abbreviations are used in the article: Tol—toluene, CHCl_3_—chloroform, DCM—dichloromethane, MeOH—methanol, MeCN—acetonitrile.

## 4. Conclusions

As a result of the conducted studies, six dibenzofulvene derivatives containing selected N-donor substituents in their structure (A-1–A-6) were obtained. All compounds were synthesized via the simple Knoevenagel condensation. The considered derivatives were purified by column chromatography. The compounds tested in the A-1–A-6 group showed one distinct absorption band in the 299–414 nm range. It was shown that the change of the substituent in position 9 affects the position of the absorption band in the entire group of compounds. Compound A-5 was the most blue-shifted. It can be concluded that this is due to oxygen in the morpholine ring. Compounds A-2 and A-3 are the most red-shifted. Comparing the obtained group of dibenzofulvene derivatives with fluorene (A-0), it can be seen that the change in the structure of the compounds significantly affects the bathochromic shift of the absorption bands (approximately 76 nm). Moreover, A-0–A-6 did not show solvatochromism during the studies that were conducted. Moreover, dibenzofulvene derivatives did not show emission in solution, unlike A-0. Electrochemical tests showed significant facilitation of the electrode processes in the A-1–A-6 group, compared to A-0. The oxidation process for the entire group was thermodynamically reversible, which proves the high stability of the molecule after p-doping. The easiest to oxidize was A-2 (0.18 V) and the most difficult was A-6 (0.42 V). In the case of the reduction process, an opposite trend was observed. Substituents influencing the facilitation of the oxidation process hindered the reduction process. Experimental studies were enriched with theoretical results. As shown, the group of A-1–A-6 derivatives are characterized by a non-planar configuration of structures. On analyzing the distribution of frontier orbitals, we observe that the HOMO electron clouds are located mainly on the N-donor substituent. On the other hand, the LUMO electron clouds are dominated by the dibenzofulvene fragment. Modifying fluorene to substituted dibenzofulvene significantly influenced the obtained IP and EA values. The obtained energy gap values for A-1–A-6 (from 2.13 eV to 2.80 eV) were lower than for A-0 (4.90 eV). This makes the A-1–A-6 molecules promising in terms of application. Interestingly, the A-2 derivative was characterized by the smallest energy gap, and the A-5 compound by the largest one.

## Figures and Tables

**Table 1:** Structures, yields, and photos of derivatives A-1–A-6.

**Code**	A-1	A-2	A-3	A-4	A-5	A-6
**Structure**	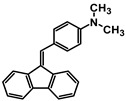	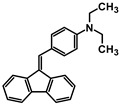	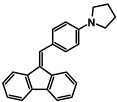	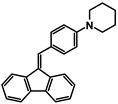	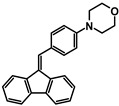	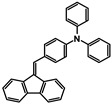
**Yield**	85%	85%	13%	90%	76%	60%
**Photo**	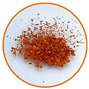	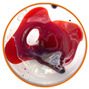	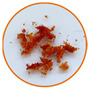	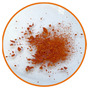	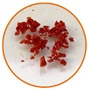	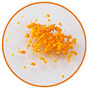

**Table 2:** Luminescence data of the tested compounds.

Compound	Structure	Solvent	λ_abs_ [nm]	ε [L·mol^−l^·cm^−l^]	PL_solid_ [nm]	Eg_opt_^1^ [eV]
A-0	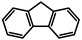	Tol	301	4774	409	4.05
		CHCl_3_	301	10,703		
		DCM	301	8810		
		ACN	299	8460		
		MeOH	300	13,096		
A-1	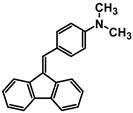	Tol	397	24,394	-	2.69
		CHCl_3_	398	23,942		
		DCM	401	21,838		
		ACN	399	23,744		
		MeOH	394	20,512		
A-2	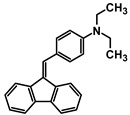	Tol	407	32,090	-	2.62
		CHCl_3_	410	27,062		
		DCM	414	20,086		
		ACN	411	28,248		
		MeOH	406	30,596		
A-3	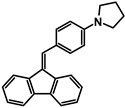	Tol	408	25,568	570	2.63
		CHCl_3_	409	25,358		
		DCM	413	20,472		
		ACN	411	25,220		
		MeOH	405	14,128		
A-4	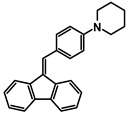	Tol	388	12,004	572	2.68
		CHCl_3_	386	22,802		
		DCM	393	19,328		
		ACN	390	24,950		
		MeOH	380	22,630		
A-5	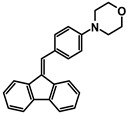	Tol	377	27,160	601	2.78
		CHCl_3_	373	23,708		
		DCM	377	16,460		
		ACN	376	21,624		
		MeOH	370	25,572		
A-6	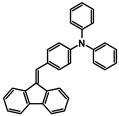	Tol	400	25,118	555	2.68
		CHCl_3_	401	23,092		
		DCM	401	23,184		
		ACN	394	22,694		
		MeOH	393	27,010		

^1^ Obtained from UV–Vis using Eg = 1240/λ_onset_ formula.

**Table 3:** IP, EA and energy gap values for A0–6.

Code	E_ox_ [V]	E_red_ [V]	IP ^1^ [eV]	EA ^1^ [eV]	Eg_(_cv_)_^2^ [eV]	IP ^3^ [eV]	EA ^3^ [eV]	Eg ^3^ [eV]	Eg ^4^ [eV]
A-0	1.30	−2.48	−6.40	−2.62	3.78	−6.22	−1.32	4.90	4.05
A-1	0.22	−2.15	−5.32	−2.95	2.37	−5.00	−2.38	2.62	2.69
A-2	0.18	−2.17	−5.28	−2.93	2.35	−4.72	−2.59	2.13	2.62
A-3	0.19	−2.19	−5.29	−2.91	2.38	−4.91	−2.35	2.55	2.63
A-4	0.24	−2.13	−5.34	−2.97	2.37	−5.12	−2.40	2.72	2.68
A-5	0.32	−2.11	−5.42	−2.99	2.42	−5.22	−2.42	2.80	2.78
A-6	0.42	−2.02	−5.52	−3.08	2.44	−5.09	−2.49	2.60	2.68

^1^ Calculated from CV measurements (IP = −5.1 − E_ox_; EA = −5.1 − E_red_); ^2^ Eg(CV) = E_ox (onset)_ − E_red (onset)_; ^3^ Obtained from DFT; ^4^ Obtained from UV–Vis using Eg = 1240/λ_onset_ formula.

## Data Availability

The original contributions presented in this study are included in the Article/Supplementary Materials. Further inquiries can be directed to the corresponding authors.

## Supplementary Materials

The following supporting information can be downloaded at: https://www.mdpi.com/article/10.3390/molecules30091924/s1, Figure S1: ^1^H NMR of A-1; Figure S2: ^13^C NMR of A-1; Figure S3: ^1^H NMR of A-2; Figure S4: ^13^C NMR of A-2; Figure S5: ^1^H NMR of A-3; Figure S6: ^13^C NMR of A-3; Figure S7: ^1^H NMR of A-4; Figure S8: ^13^C NMR of A-4; Figure S9: ^1^H NMR of A-5; Figure S10: ^13^C NMR of A-5; Figure S11: ^1^H NMR of A-6; Figure S12: ^13^C NMR of A-6; Figure S13: DSC thermograms of A-1; Figure S14: DSC thermograms of A-3; Figure S15: DSC thermograms of A-4; Figure S16: DSC thermograms of A-5; Figure S17: DSC thermograms of A-6; Figure S18: Comparison of the absorption behavior of dibenzofulvene derivatives in different solvents; Figure S19: Comparison of the absorption properties of compounds A-1–A-6 with fluorene A-0; Figure S20: HOMO, LUMO and angles plots; Figure S21: Electronic spectra of A-1–A-6 in dichloromethane (pink line) alongside with national transition orbitals (black sticks) calculated for vertical excitations, which were assigned to the lowest energy absorption band; Figure S22: Comparison of simulated UV–Vis spectra of A-1–A-6 with their N-donor substituents; Figure S23: UV–Vis spectro-electrochemistry of the A-3 derivative in MeCN solution (c = 1 × 10^−5^ mol/L, as an inset on each graph, all potentials vs. Fc/Fc^+^ redox couple.
