# Supplementary material for: The Effect of Modifying the C9 Position of Fluorene with N-Donor Substituents on Selected Physicochemical Properties
Source: Molecules. 2025 Apr 25;30(9):1924. doi: 10.3390/molecules30091924 (PMC12073123; doi:10.3390/molecules30091924)
Supplement: Supplementary file 1 [file molecules-30-01924-s001.zip › molecules-3555202-supplementary.pdf]

Supporting Information

The effect of modifying the C9 position of fluorene with N-donor substituents on selected physicochemical properties

Paweł Kalarus [a], Agata Szłapa-Kula [a], Michał Filapek [a]*, Sławomir Kula [a]*

[a] Institute of Chemistry, Faculty of Science and Technology, University of Silesia, Szkolna 9 St., 40-007 Katowice, Poland; pawel.kalarus@us.edu.pl, agata.szlapa-kula@us.edu.pl, michal.filapek@us.edu.pl, slawomir.kula@us.edu.pl,

* Correspondence: michal.filapek@us.edu.pl Tel.: +48-32-359 1646; slawomir.kula@us.edu.pl; Tel.: +48-32-359 2204

Table of Contents

1. Materials 2
2. Measurements and general methods 2
3. Synthesis and NMR spectra 3
4. Thermal properties 12
5. Optical properties 13
6. DFT calculations 16
7. Electrochemistry 22
8. Literature 22

1. Materials

Fluorene (98+%, Thermo Scientific), Potassium tert-butoxide (>97.0%, TCI), Ethyl alcohol (96%, POCH), 4-Dimethylaminobenzaldehyde (>99.0%, Fluka Chemika), 4-Diethylaminobenzaldehyde (99%, Merck-Sigma-Aldrich), 4-Pyrrolidinobenzaldehyde (98%, Combi-Blocks), 4-(Piperidin-1-yl)benzaldehyde (>97%, Alfa Chemical), 4-Morpholinobenzaldehyde (98%, Combi-Blocks), 4-(N,N-Diphenylamino)benzaldehyde (>98.0%, TCI), Chloroform-d ($CDCl_3$, 99.8 atom %D, Merck-Sigma-Aldrich), Ethyl acetate (p.a., POCH), Hexane (p.a., Stanlab), Dichloromethane (99.5%, PureLand), Acetonitrile (for HPLC-GC, ≥99.8% (GC), Merck-Sigma-Aldrich), Chloroform (for HPLC, ≥99.8%, amylene stabilized, Merck-Sigma-Aldrich), Methanol (for HPLC, ≥99.9%, Merck-Sigma-Aldrich), Toluene (pure for analysis, Chempur). Thin Layer Chromatography (Merck TLC Silica Gel 60). Silica gel from Merck was used for column chromatography.

2. Measurements and general methods

Bruker Avance 500 instrument were used to record the NMR spectra in $CDCl_3$ (as a solvent). UV/Vis spectra were recorded with a Biosens model UV 5600 UV/Vis spectrophotometer. Photoluminescence emission spectra were acquired using Hitachi Fluorescence Spectrophotometer F-7100. Differential scanning calorimetry (DSC) was performed on a TA-DSC 25 device. Electrochemical measurements were carried out with an Eco Chemie Autolab PGSTAT128n potentiostat using glassy carbon (with diam. 2 mm) as working electrode, while platinum coil and silver wire were used as auxiliary and reference electrode, respectively. Potentials are referenced with respect to ferrocene (Fc), which was used as the internal standard. Cyclic and differential pulse voltammetry experiments were conducted in a standard one-compartment cell, in acetonitrile (MeCN) (Carlo Erba, HPLC grade), under argon. Bu_4NPF_6 (Aldrich; 0.2 M, 99%) was used as the supporting electrolyte. UV–Vis spectro-electrochemical measurements were performed in a 1 cm quartz cuvette with indium tin oxide (ITO, with 10 Ω per square) glass working electrode; platinum and silver wire were used as auxiliary and reference electrodes, respectively.

3. Synthesis and NMR spectra

General synthesis procedure for compounds A-1 – A-6

A mixture of fluorene (4.00 mmol), potassium tert-butoxide (4.00 mmol), and ethanol (10 mL) was heated at reflux for 1 h, under inert gas atmosphere (argon). Then, the selected aldehyde (12.00 mmol) was added to the reaction mixture in portions, and heating was continued for another 24 h. After 24 h, the reaction mixture was extracted using dichloromethane and an aqueous saturated sodium chloride solution. The obtained organic fraction was evaporated in a vacuum evaporator. The crude product was purified by column chromatography. The synthetic procedure was developed based on modifications of selected literature methods [1].

9-(4-(N,N-dimethylamino)benzylidene)-9H-fluorene (A-1)

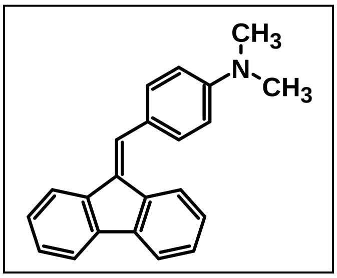

The aldehyde used in this reaction was 4-dimethylaminobenzaldehyde. The crude product was purified by double column chromatography using silica gel as the solid phase and a mixed solvent of hexane:ethyl acetate (10:1) as the mobile phase. **A-1** was obtained as a dark orange solid in 85% yield. Melting point: 137 °C (Figure S13). ^{1}H NMR (500 MHz, $CDCl_3$) δ 7.99 (d, J = 7.9 Hz, 1H), 7.84 – 7.79 (m, 1H), 7.79 – 7.74 (m, 2H), 7.68 (s, 1H), 7.59 (d, J = 8.4 Hz, 2H), 7.40 – 7.31 (m, 3H), 7.19 – 7.12 (m, 1H), 6.82 (d, J = 6.6 Hz, 2H), 3.09 (s, 6H). ^{13}C NMR (125 MHz, $CDCl_3$) δ 140.82, 140.15, 138.57, 136.85, 134.71, 133.44, 131.10, 129.10, 128.62, 127.81, 127.32, 126.72, 126.46, 123.98, 119.87, 119.63, 119.45, 111.82, 40.41.

9-(4-(N,N-diethylamino)benzylidene)-9H-fluorene (A-2)

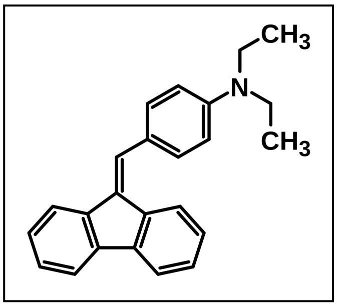

The aldehyde used in this reaction was 4-(N,N-diphenylamino)benzaldehyde. The crude product was purified by double column chromatography using silica gel as the solid phase and a mixed solvent of hexane:ethyl acetate (5:1) as the mobile phase. **A-2** was obtained as a thick, dark red oil in 85% yield. Melting point: - . ^{1}H NMR (500 MHz, $CDCl_3$) δ 8.09 (d, J = 7.8 Hz, 1H), 7.81 (d, J = 6.7 Hz, 1H), 7.77 (t, J = 6.9 Hz, 2H), 7.67 (s, 1H), 7.59 (d, J = 8.2 Hz, 2H), 7.38 – 7.32 (m, 3H), 7.19 – 7.15 (m, 1H), 6.76 (d, J = 8.9 Hz, 2H), 3.47 (q, J = 7.2 Hz, 4H), 1.27 (t, J = 7.1 Hz, 6H). ^{13}C NMR (125 MHz, $CDCl_3$) δ 147.80, 140.71, 140.32, 138.42, 136.91,

132.72, 131.50, 128.91, 127.63, 127.11, 126.66, 126.40, 123.96, 123.13, 119.78, 119.60, 119.42, 111.04, 44.42, 12.71.

9-(4-(pyrrolidin-4-yl)benzylidene)-9H-fluorene (A-3)

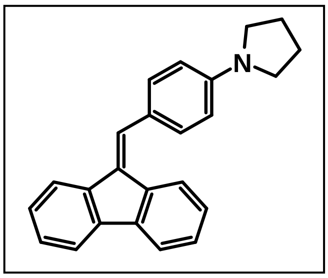

The aldehyde used in this reaction was 4-pyrrolidinobenzaldehyde. The crude product was purified by double column chromatography using silica gel as the solid phase and a mixed solvent of hexane:ethyl acetate (ratios, respectively: 5:1, 10:1) as the mobile phase. **A-3** was obtained as a dark orange crystalline solid in 13% yield. Melting point: 179 ºC (Figure S14). ^{1}H NMR (500 MHz, $CDCl_3$) δ 8.03 (d, *J* = 7.9 Hz, 1H), 7.81 (dd, *J* = 6.5, 1.8 Hz, 1H), 7.79 – 7.74 (m, 2H), 7.69 (s, 1H), 7.59 (d, *J* = 8.5 Hz, 2H), 7.39 – 7.31 (m, 3H), 7.17 – 7.13 (m, 1H), 6.69 (d, *J* = 6.7 Hz, 2H), 3.44 – 3.41 (m, 4H), 2.11 – 2.06 (m, 4H). ^{13}C NMR (125 MHz, $CDCl_3$) δ 140.74, 140.24, 138.47, 136.89, 131.32, 127.68, 127.17, 126.67, 126.42, 123.91, 119.80, 119.61, 119.43, 29.72, 25.51.

9-(4-(piperidin-1-yl)benzylidene)-9H-fluorene (A-4)

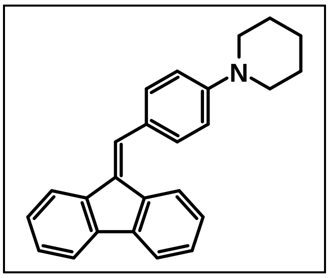

The aldehyde used in this reaction was 4-(piperidin-1-yl)benzaldehyde. The crude product was purified by double column chromatography using silica gel as the solid phase and a hexane:ethyl acetate (ratios, respectively: 10:1, 1:1) as the mobile phase. **A-4** was obtained as a brown solid in 90% yield. Melting point: 119 ºC (Figure S15). ^{1}H NMR (500 MHz, $CDCl_3$) δ 7.92 (d, *J* = 8.1 Hz, 1H), 7.80 (d, *J* = 6.6 Hz, 1H), 7.77 – 7.74 (m, 2H), 7.66 (s, 1H), 7.57 (d, *J* = 8.7 Hz, 2H), 7.40 – 7.31 (m, 3H), 7.15 – 7.11 (m, 1H), 7.06 – 6.95 (m, 2H), 3.32 (t, *J* = 5.5 Hz, 4H), 1.83 – 1.63 (m, 6H). ^{13}C NMR (125 MHz, $CDCl_3$) δ 140.95, 140.03, 138.74, 136.80, 134.25, 130.88, 128.42, 128.18, 128.02, 127.57, 126.81, 126.54, 124.17, 119.98, 119.67, 119.51, 115.35, 49.88, 25.71, 24.33.

9-(4-(morpholin-4-yl)benzylidene)-9H-fluorene (A-5)

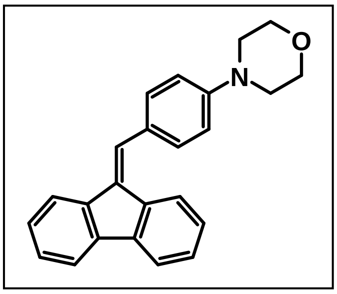

The aldehyde used in this reaction was 4-morpholinobenzaldehyde. The crude product was purified by double column chromatography using silica gel as the solid phase and a mixed solvent of hexane:ethyl acetate (1:1) as the mobile phase. **A-5** was obtained as a dark red solid in 76% yield. Melting point: 177 °C (Figure S16). ^{1}H NMR (500 MHz, $CDCl_3$) δ 7.80 (d, J = 6.6 Hz, 2H), 7.77 – 7.72 (m, 2H), 7.65 (s, 1H), 7.61 (d, J = 8.7 Hz, 2H), 7.41 – 7.37 (m, 1H), 7.37 – 7.32 (m, 2H), 7.15 – 7.10 (m, 3H), 4.04 – 3.92 (m, 4H), 3.35 (t, J = 5.0 Hz, 4H). ^{13}C NMR (125 MHz, $CDCl_3$) δ 141.12, 139.74, 138.90, 136.58, 131.68, 130.91, 128.88, 128.32, 127.90, 126.91, 126.59, 124.13, 120.07, 119.75, 119.56, 67.15, 51.64.

9-(4-(N,N-diphenylamino)benzylidene)-9H-fluorene (A-6)

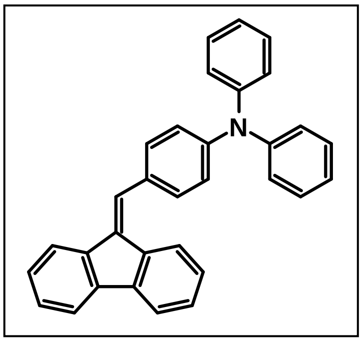

The aldehyde used in this reaction was 4-(N,N-diphenylamino)benzaldehyde. The crude product was purified by double column chromatography using silica gel as a solid phase and a mixed solvent of hexane:ethyl acetate (ratios, respectively: 5:1, 10:1) as a mobile phase. **A-6** was obtained as a yellow solid in 60% yield. Melting point: 116 °C (Figure S17). ^{1}H NMR (500 MHz, $CDCl_3$) δ 7.92 (d, J = 7.9 Hz, 1H), 7.82 – 7.78 (m, 1H), 7.75 (dd, J = 7.6, 3.9 Hz, 2H), 7.65 (s, 1H), 7.54 (d, J = 8.1 Hz, 2H), 7.41 – 7.35 (m, 2H), 7.35 – 7.31 (m, 5H), 7.21 (dd, J = 8.6, 1.1 Hz, 4H), 7.16 (t, J = 9.0 Hz, 3H), 7.13 – 7.08 (m, 2H). ^{13}C NMR (125 MHz, $CDCl_3$) δ 147.86, 147.41, 141.13, 139.84, 138.86, 136.59, 135.15, 130.64, 130.07, 129.42, 128.29, 127.84, 127.41, 126.89, 126.60, 124.96, 124.24, 123.46, 122.43, 120.05, 119.74, 119.54.

NMR spectra

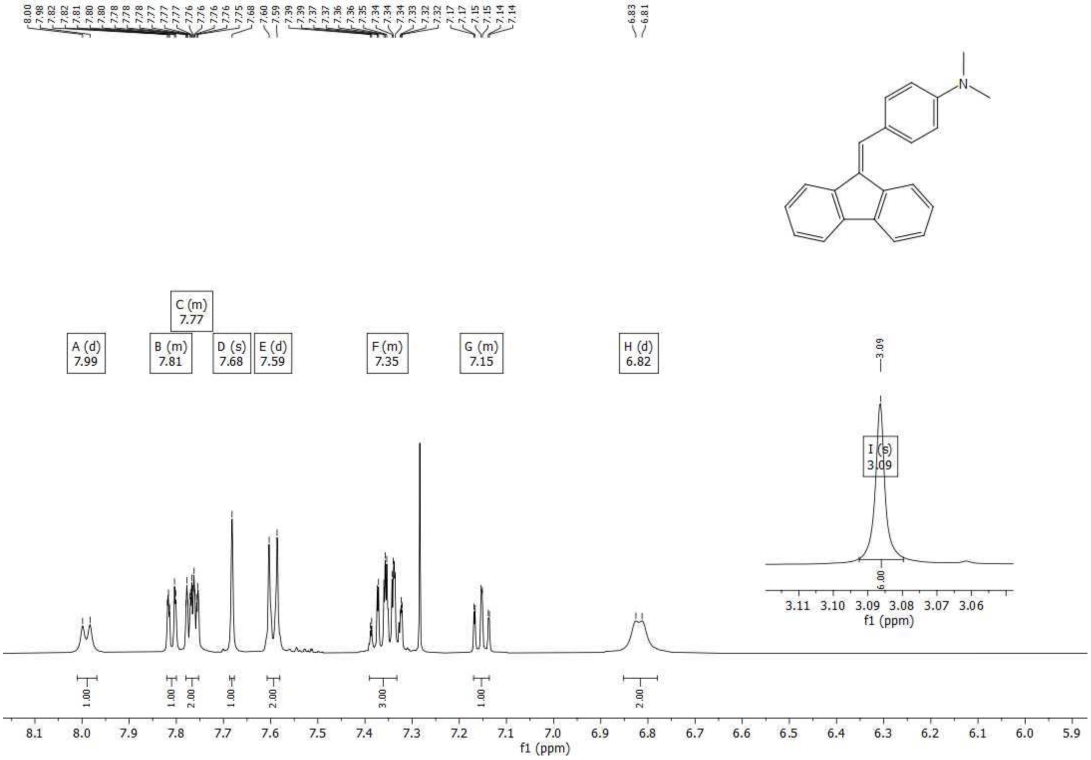

Figure S1. ^{1}H NMR of A-1

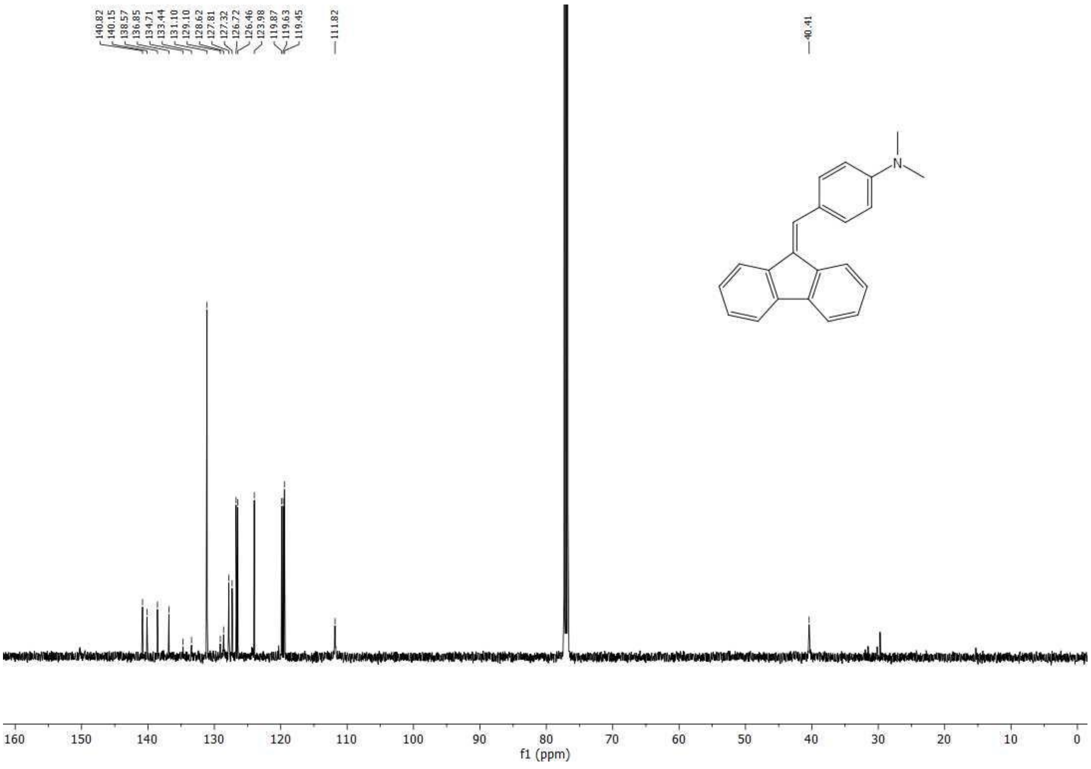

Figure S2. ^{13}C NMR of A-1

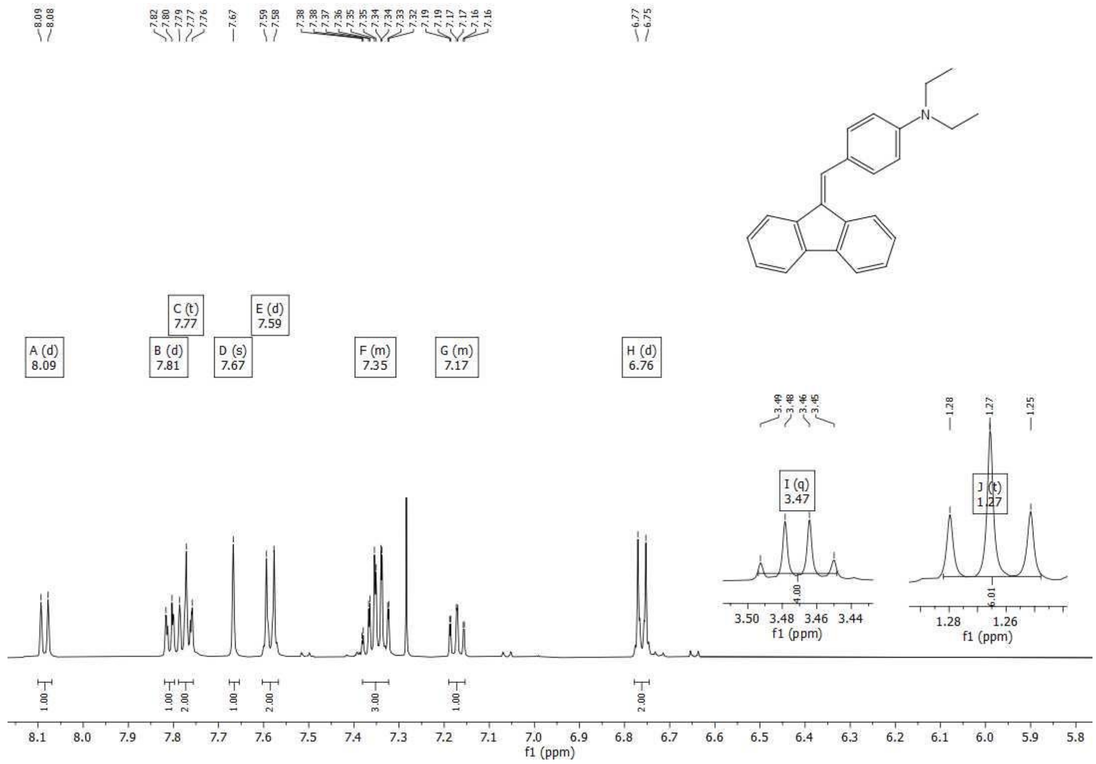

Figure S3. ^{1}H NMR of A-2

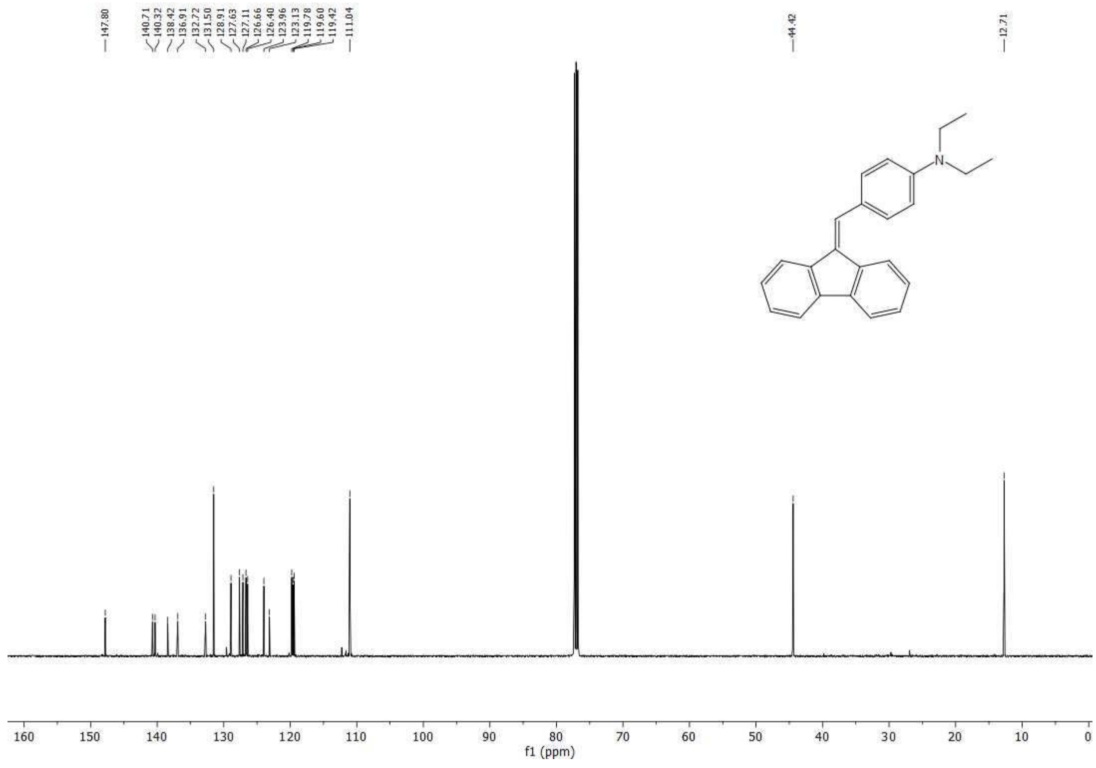

Figure S4. ^{13}C NMR of A-2

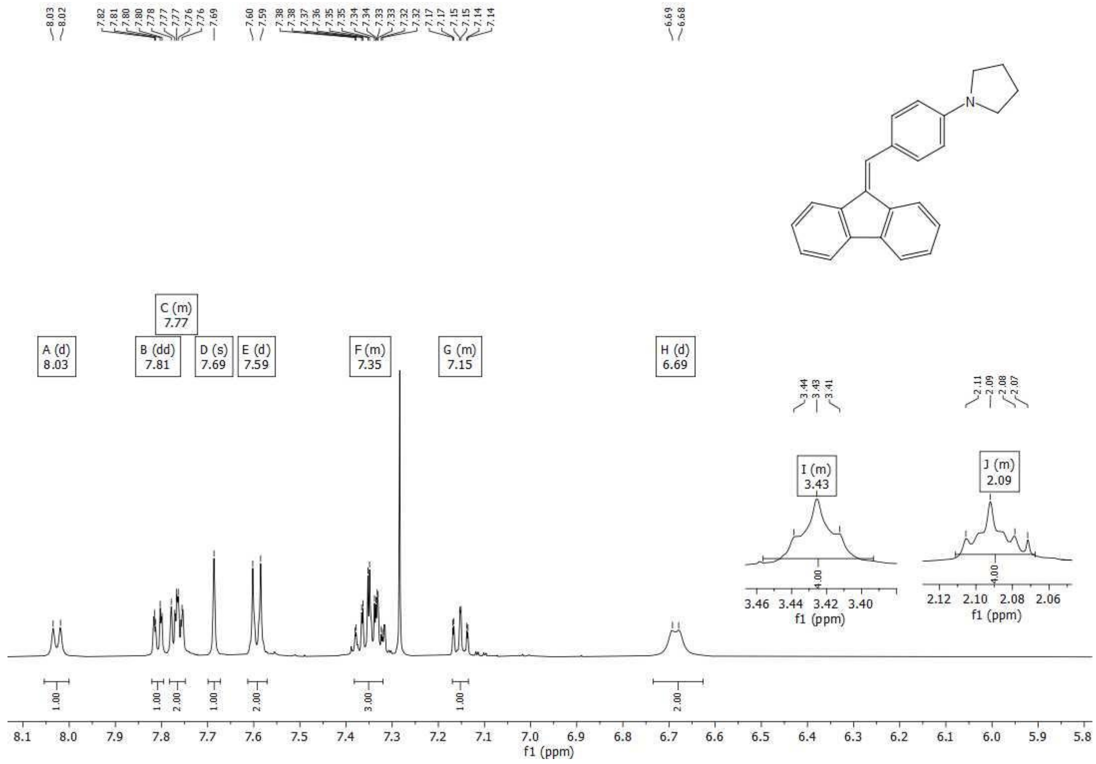

Figure S5. ^{1}H NMR of A-3

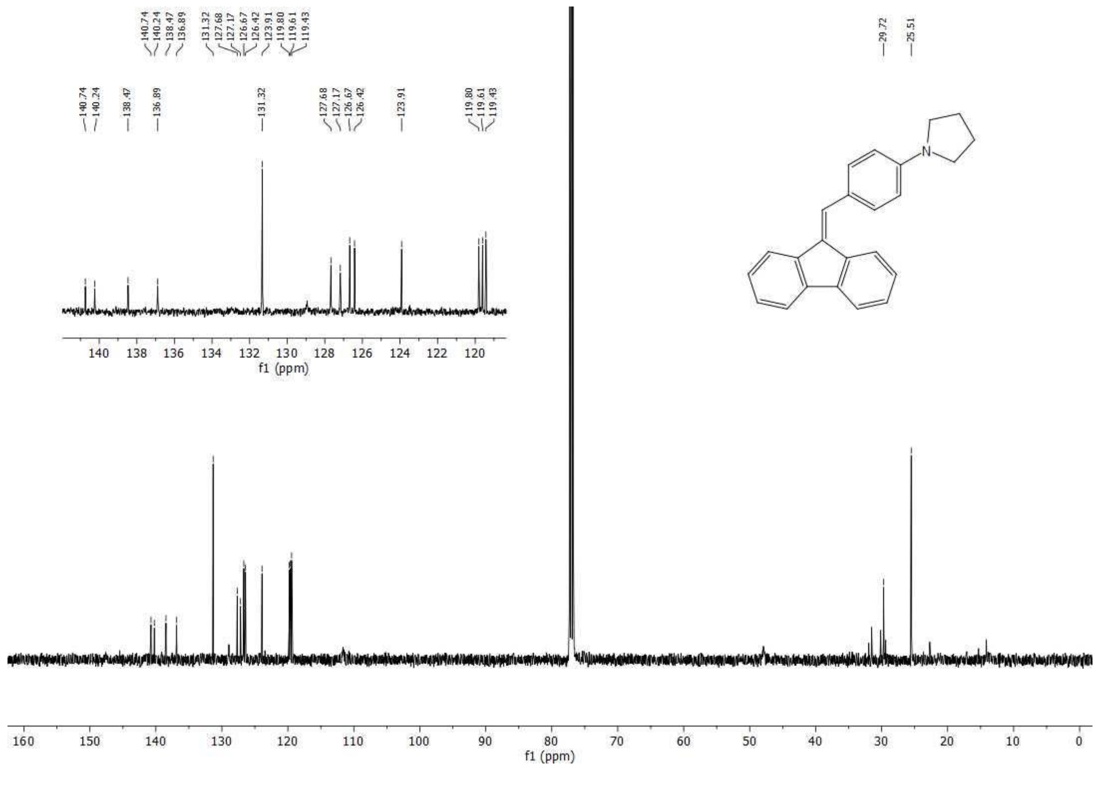

Figure S6. ^{13}C NMR of A-3

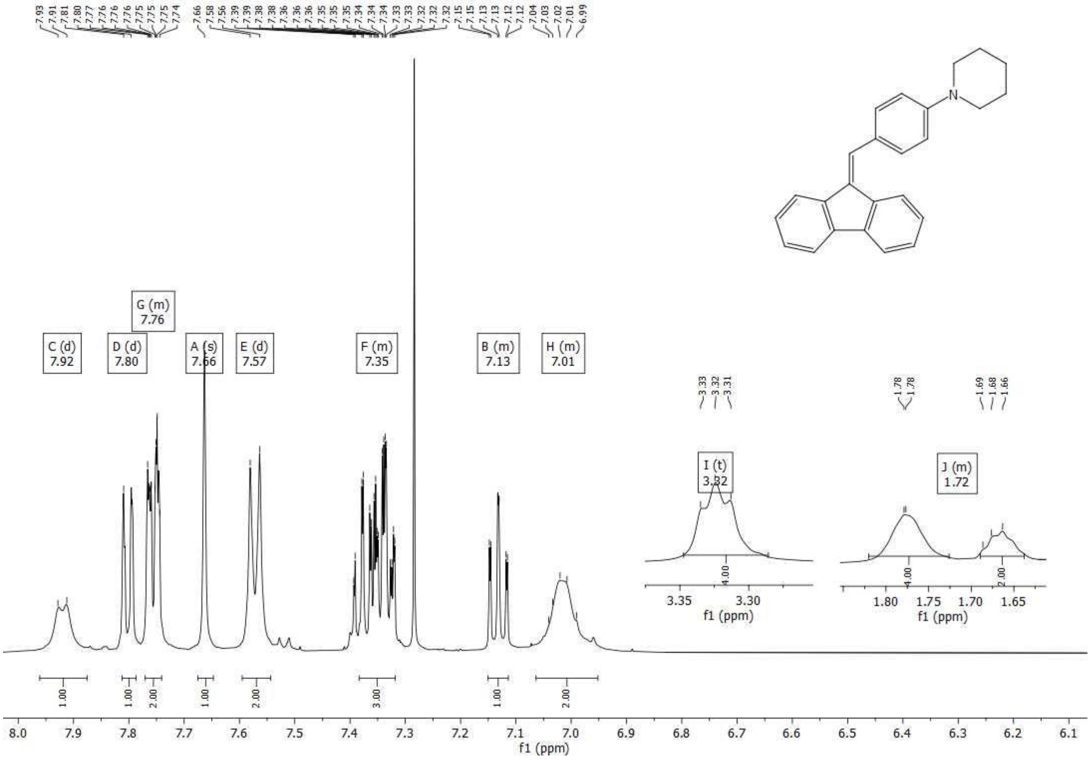

Figure S7. ^{1}H NMR of A-4

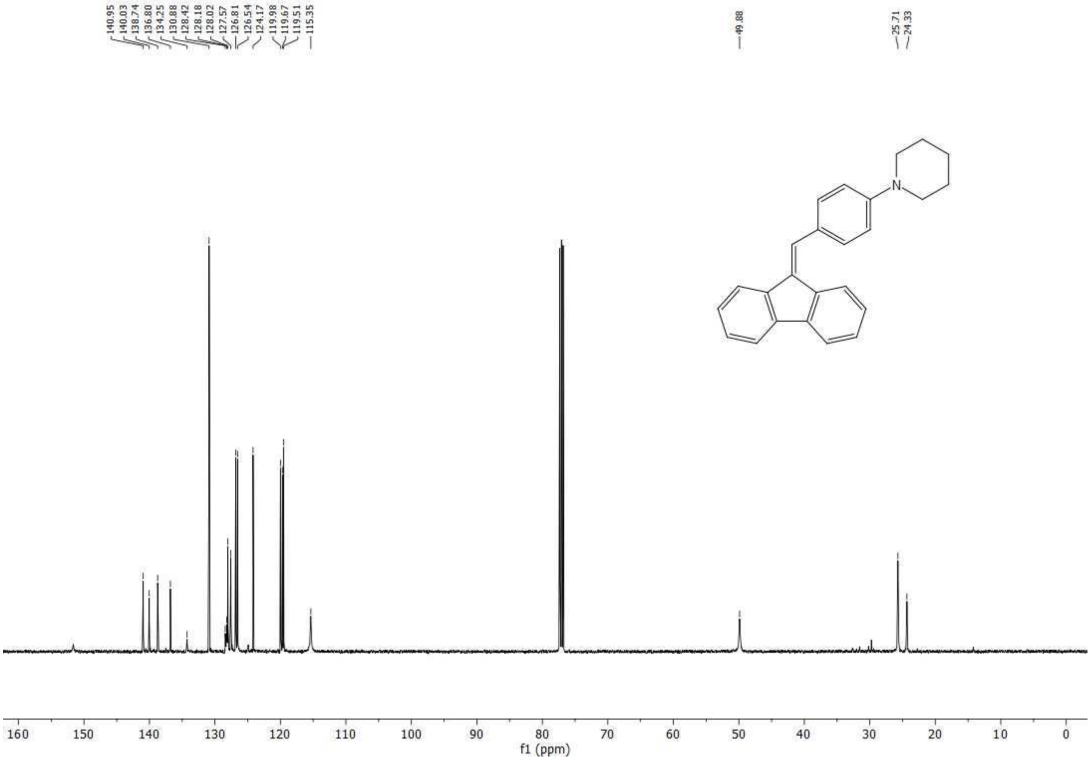

Figure S8. ^{13}C NMR of A-4

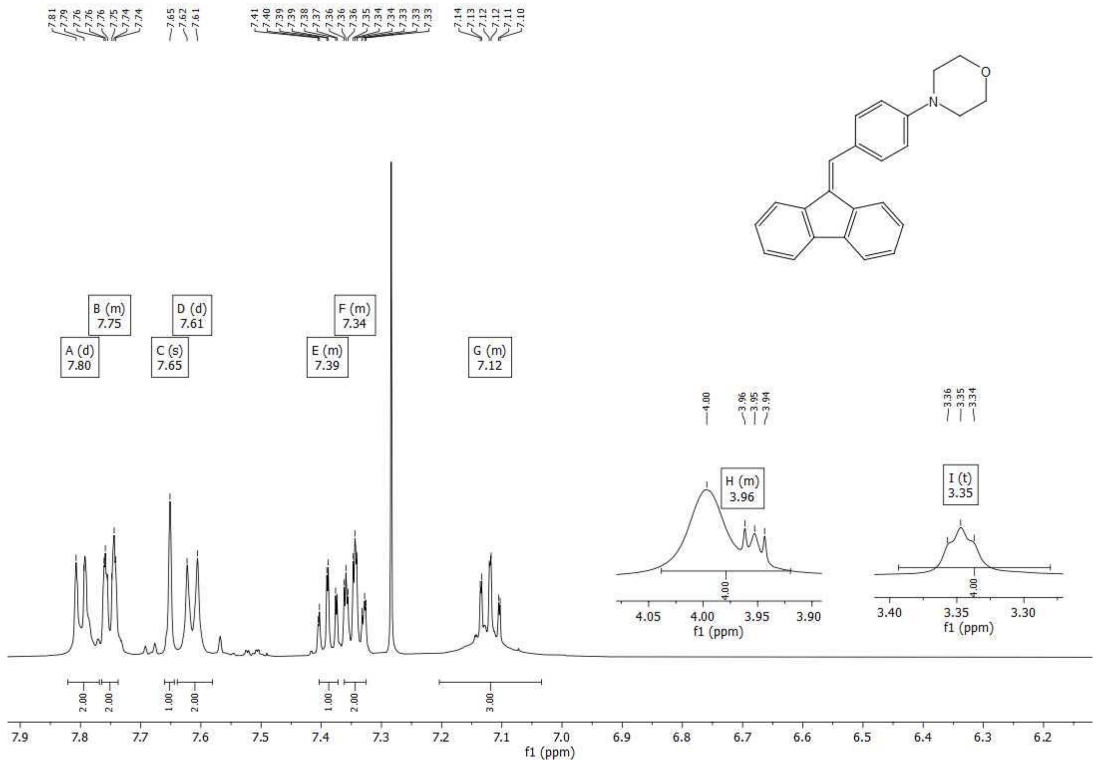

Figure S9. ^{1}H NMR of A-5

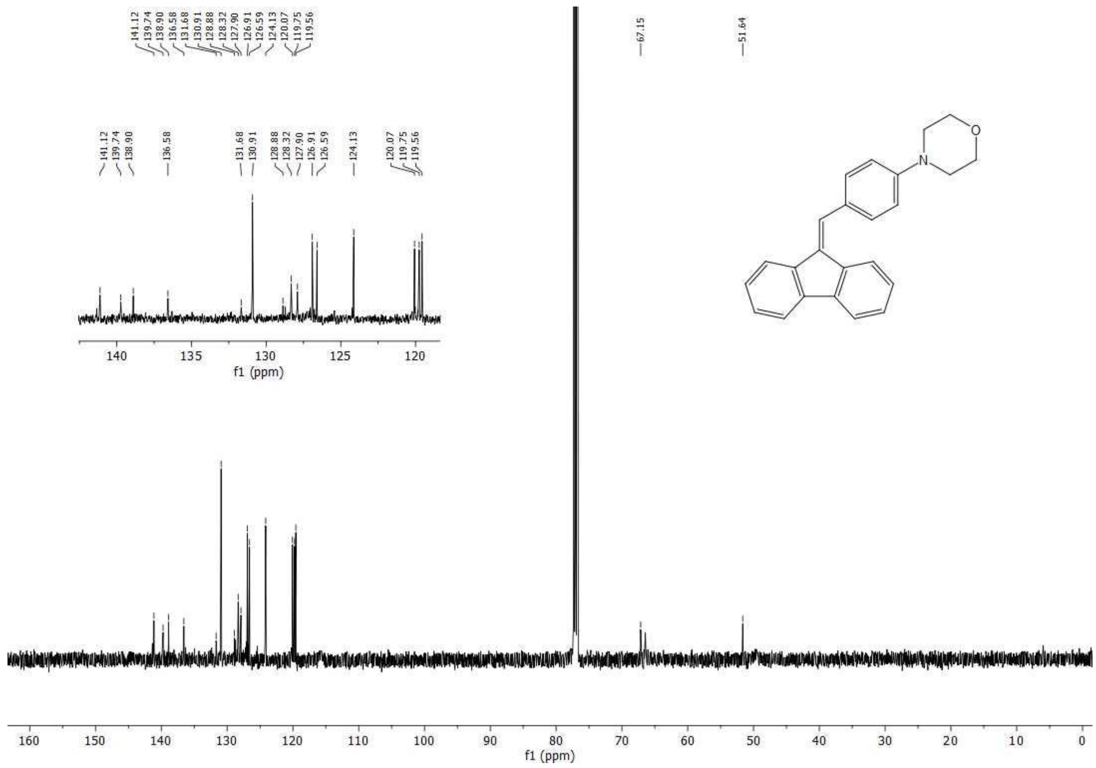

Figure S10. ^{13}C NMR of A-5

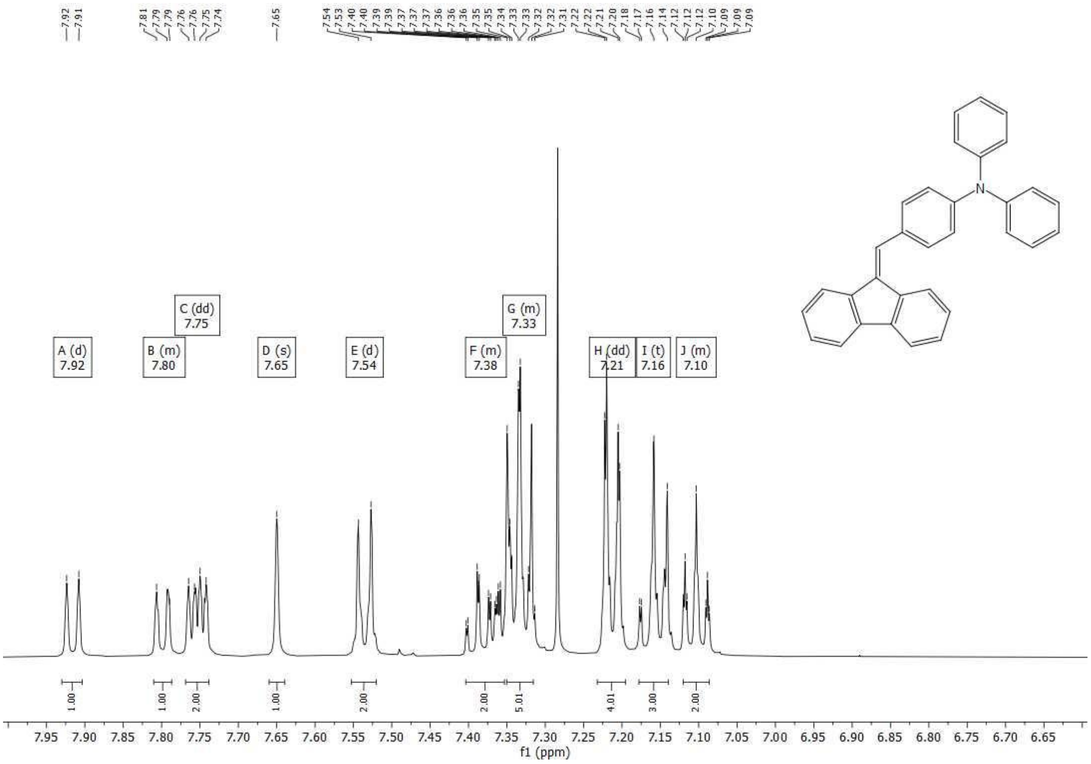

Figure S11. ^{1}H NMR of A-6

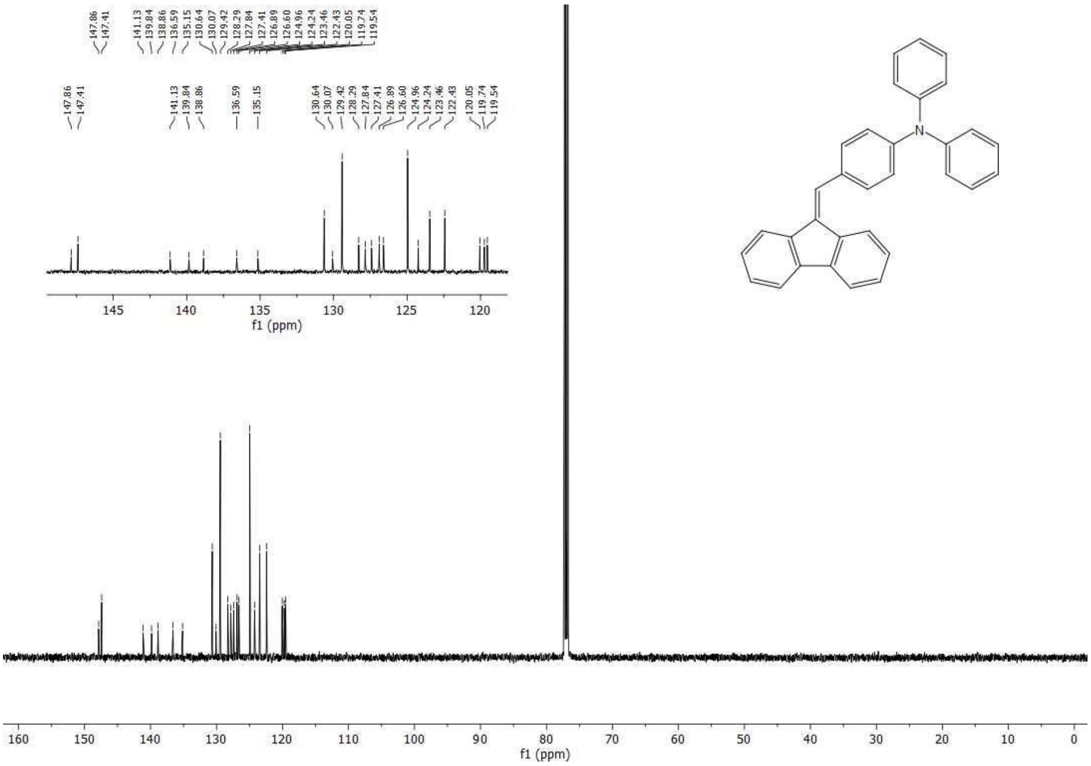

Figure S12. ^{13}C NMR of A-6

4. Thermal properties

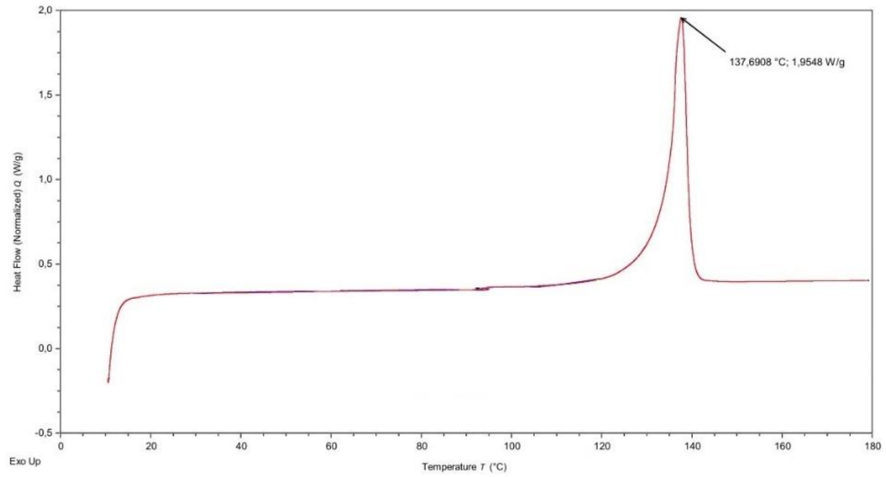

Figure S13. DSC thermograms of **A-1**

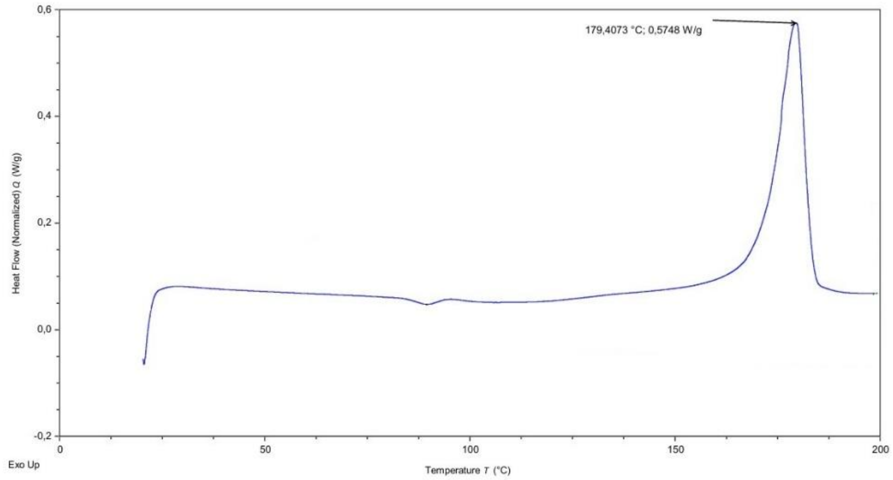

Figure S14. DSC thermograms of **A-3**

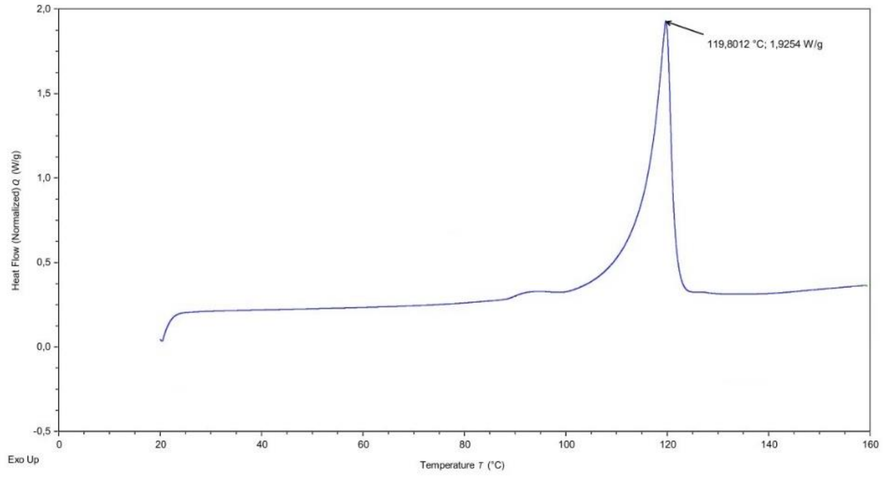

Figure S15. DSC thermograms of **A-4**

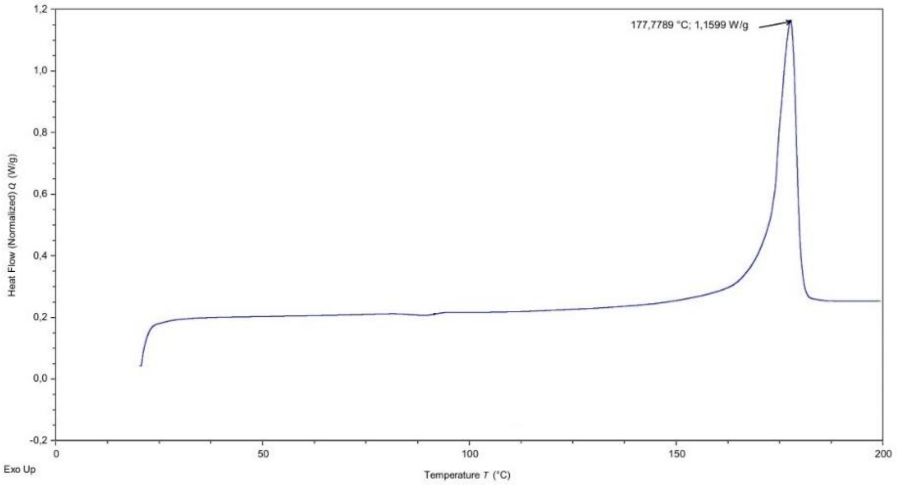

Figure S16. DSC thermograms of **A-5**

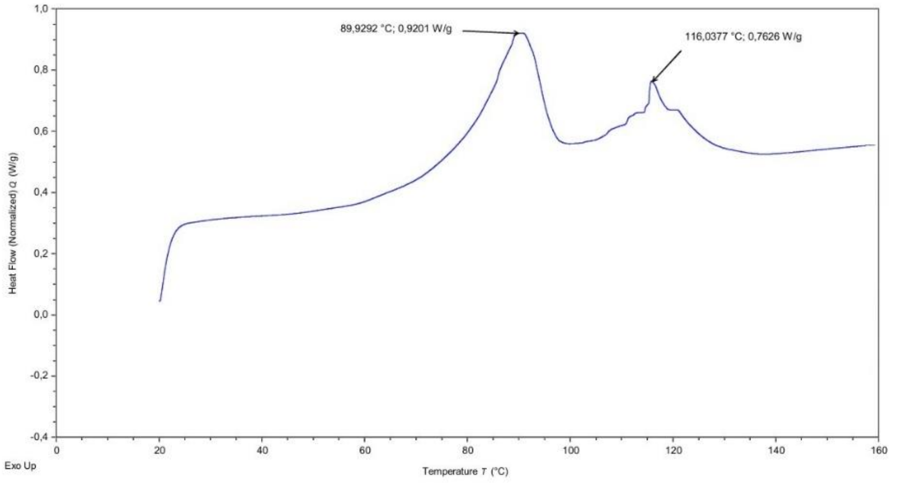

Figure S17. DSC thermograms of **A-6**

5. Optical properties

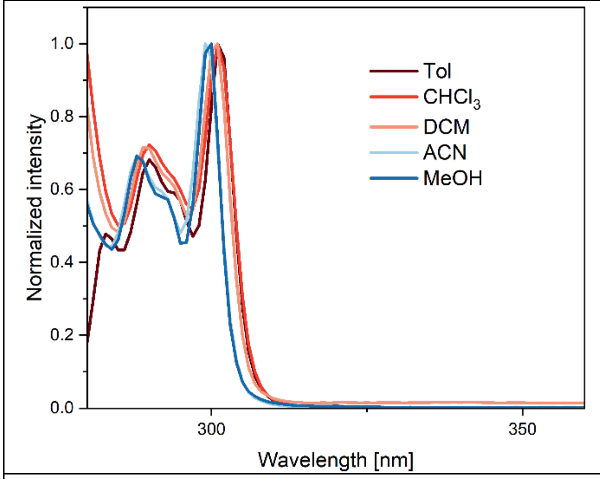	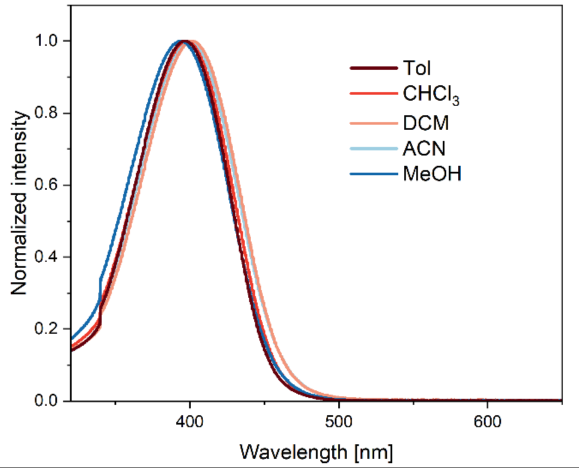
**A-0**	**A-1**

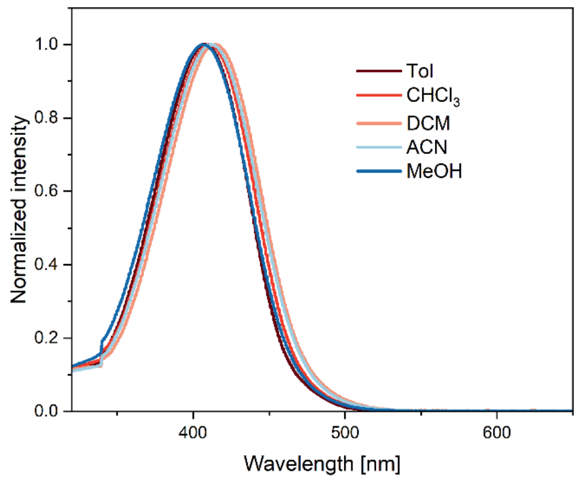

A-2

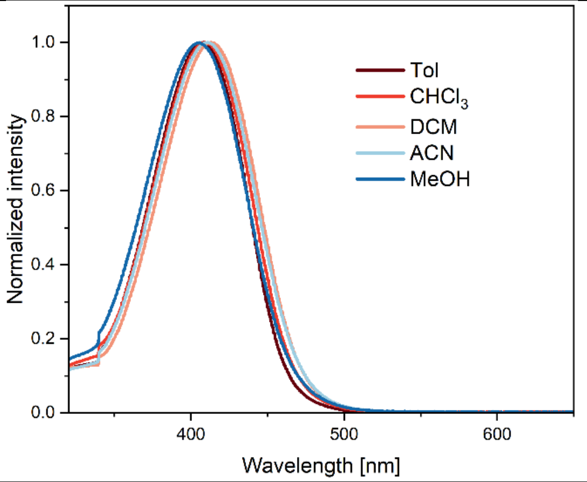

A-3

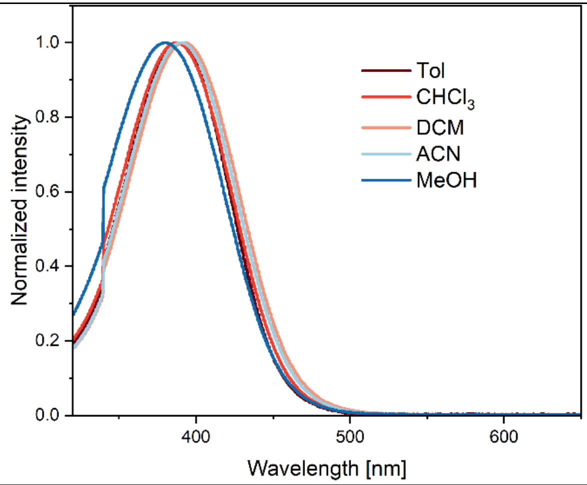

A-4

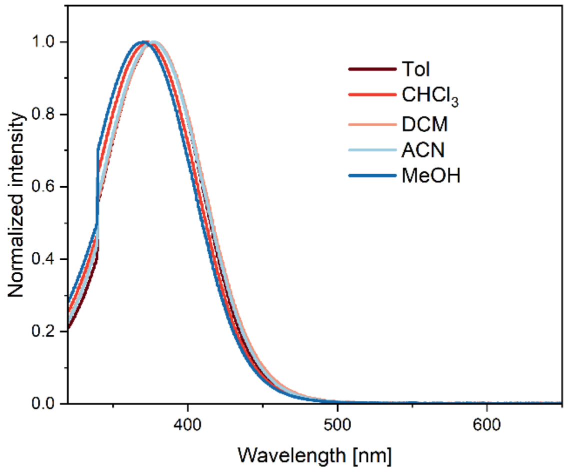

A-5

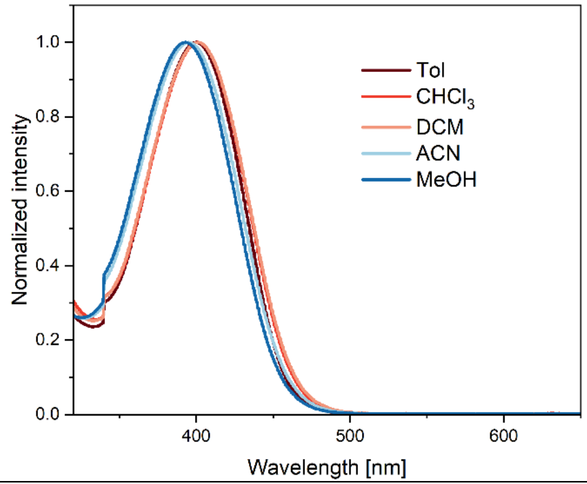

A-6

Figure S18. Comparison of the absorption behavior of dibenzofulvene derivatives in different solvents

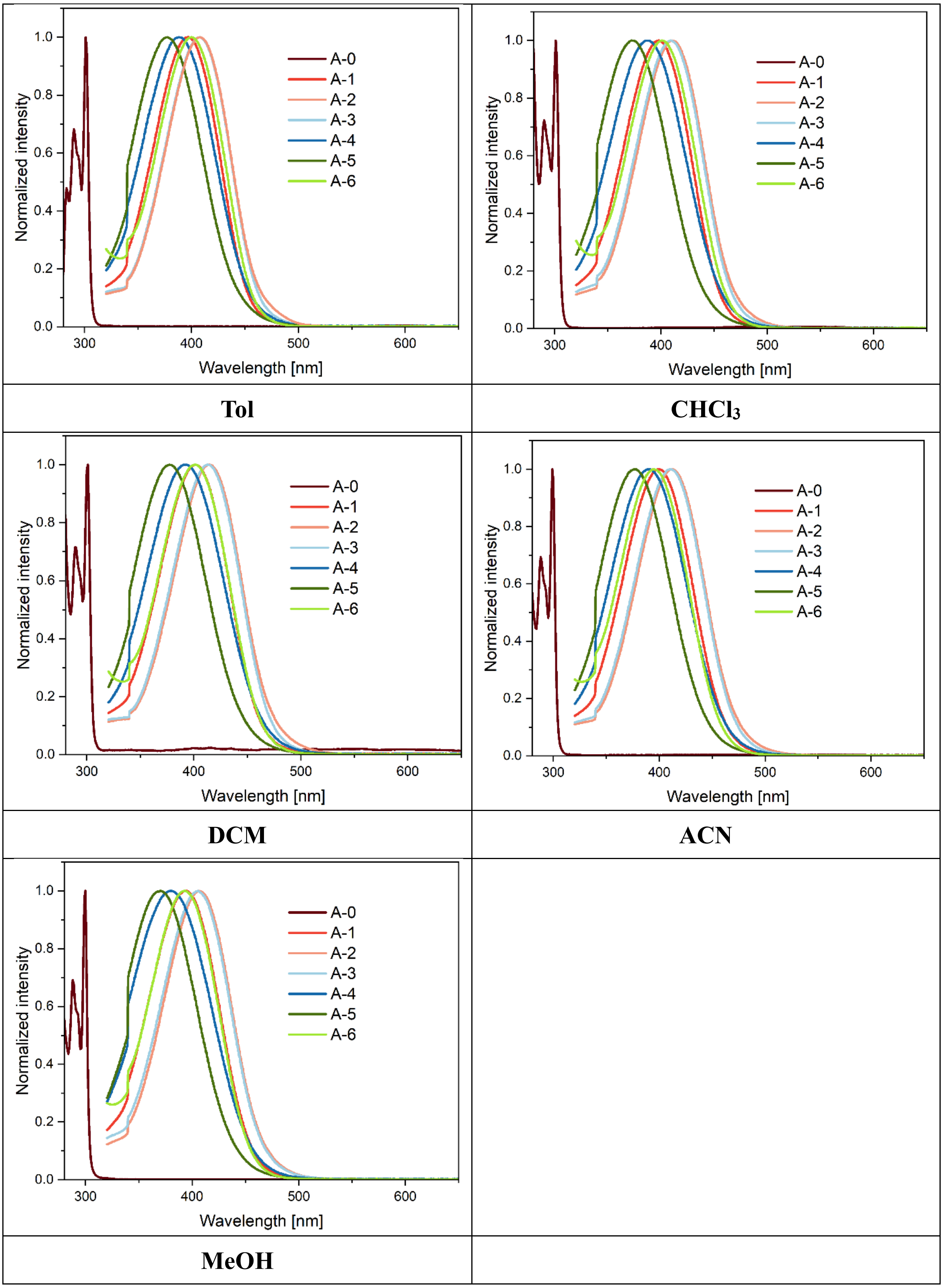

Figure S19. Comparison of the absorption properties of compounds A-1–A-6 with fluorene A-0

6. DFT calculations

No.	HOMO	LUMO	Angles
**A-0**	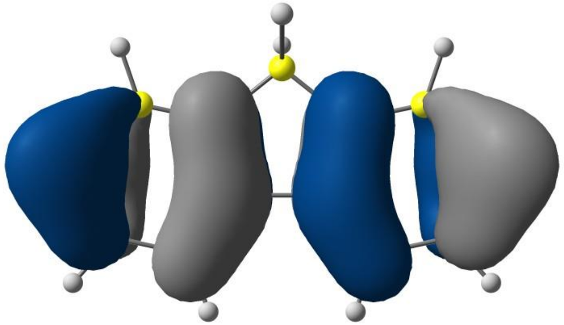	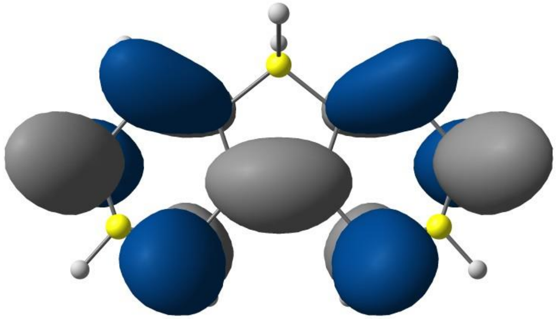	-
**A-1**	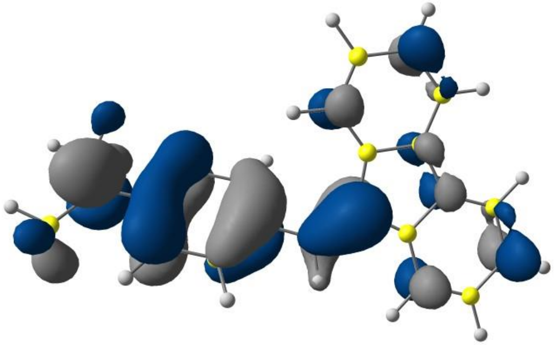	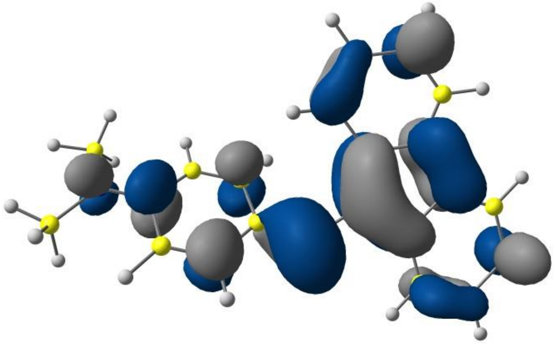	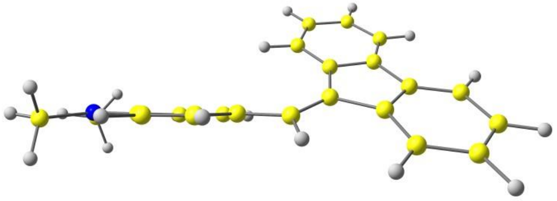
**A-2**	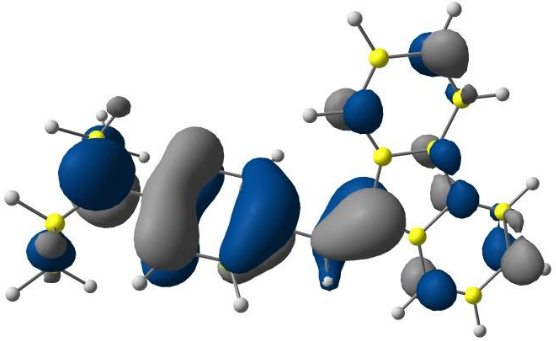	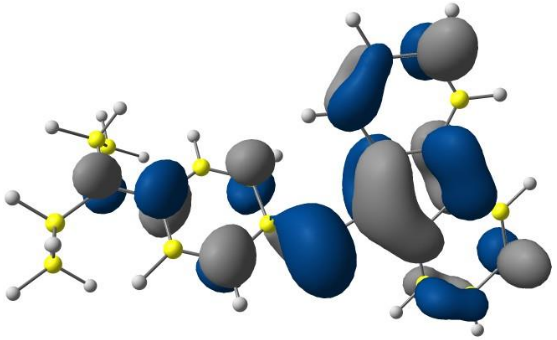	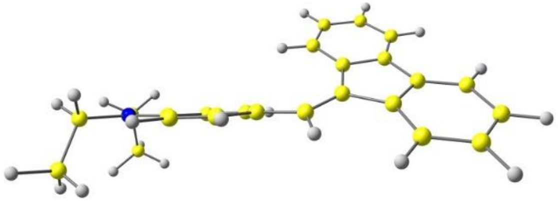

**A-3**	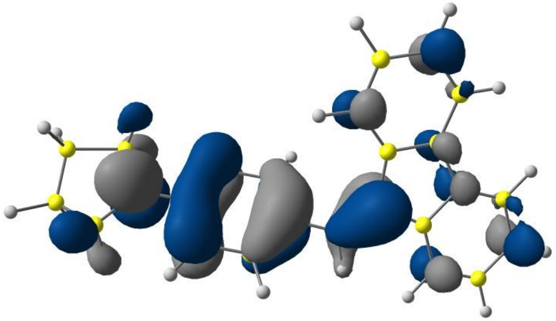	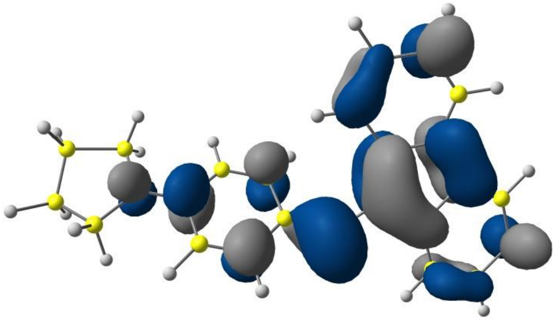	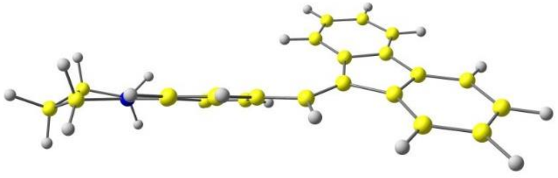
**A-4**	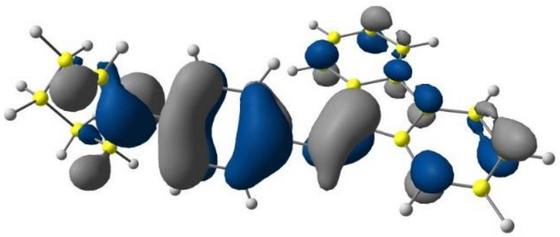	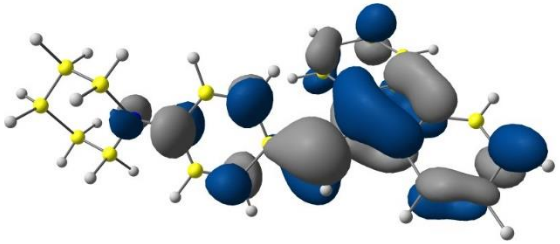	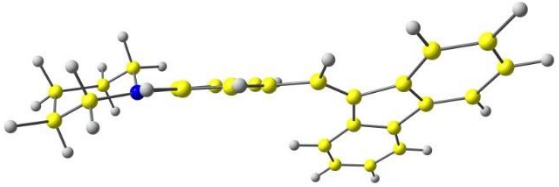
**A-5**	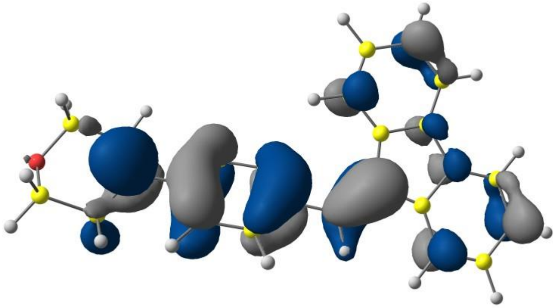	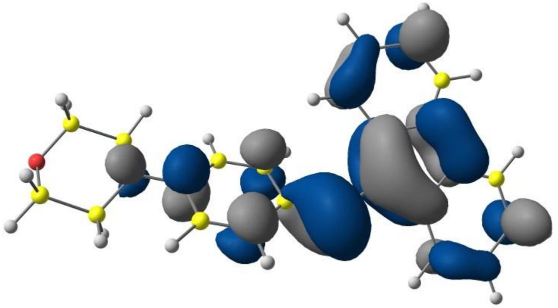	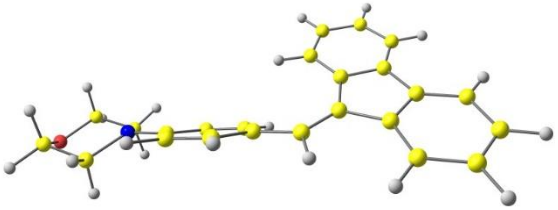

**A-6**	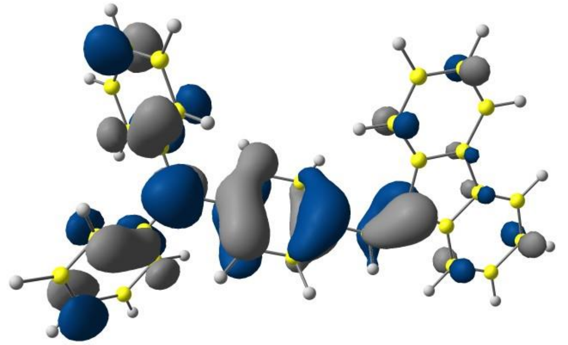	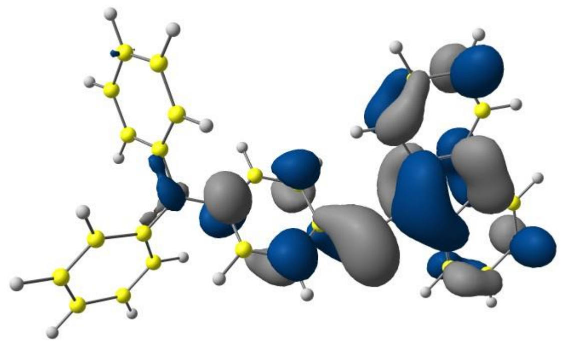	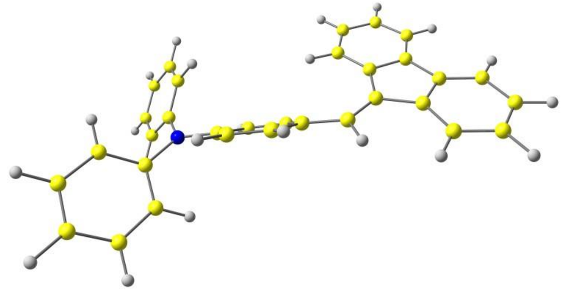

Figure S20. HOMO, LUMO and angles plots

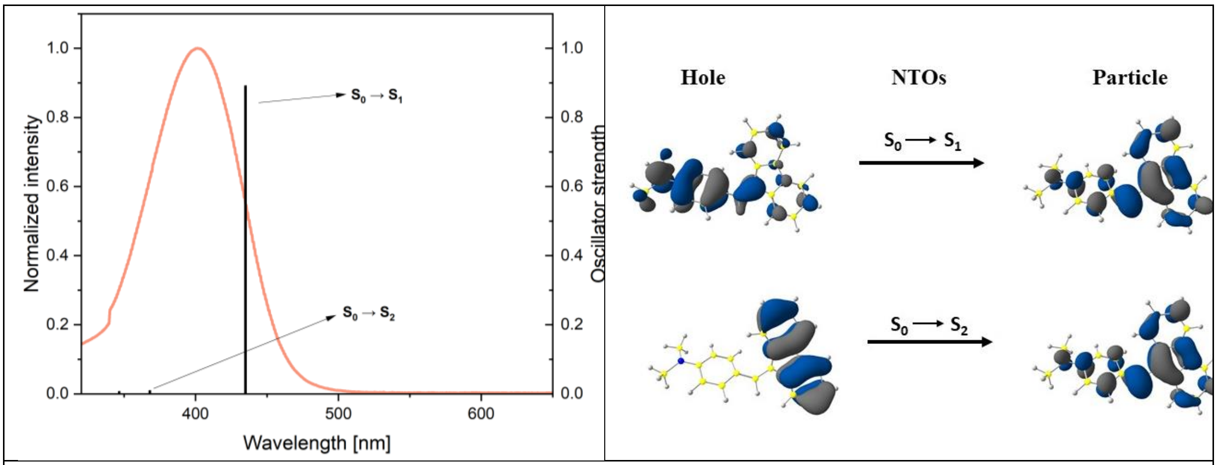

Normalized intensity
Oscillator strength
Wavelength [nm]
$S_0 \rightarrow S_1$
$S_0 \rightarrow S_2$
Hole
NTOs
Particle

A-1

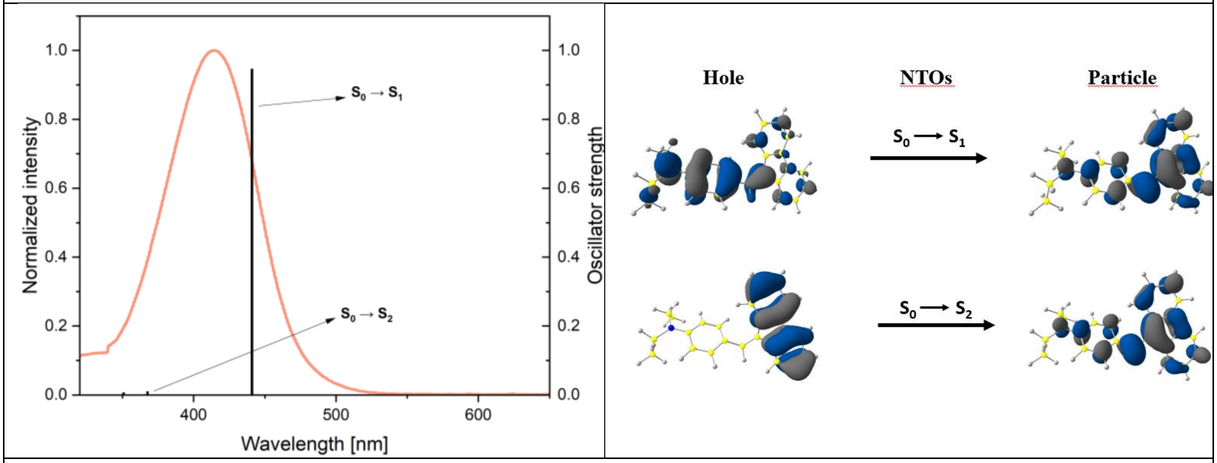

Normalized intensity
Oscillator strength
Wavelength [nm]
$S_0 \rightarrow S_1$
$S_0 \rightarrow S_2$
Hole
NTOs
Particle

A-2

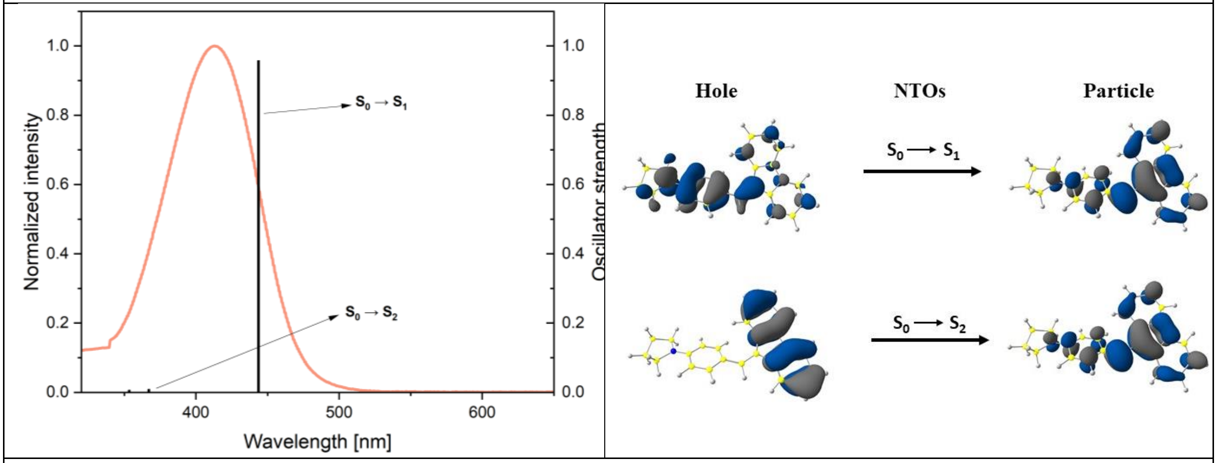

Normalized intensity
Oscillator strength
Wavelength [nm]
$S_0 \rightarrow S_1$
$S_0 \rightarrow S_2$
Hole
NTOs
Particle

A-3

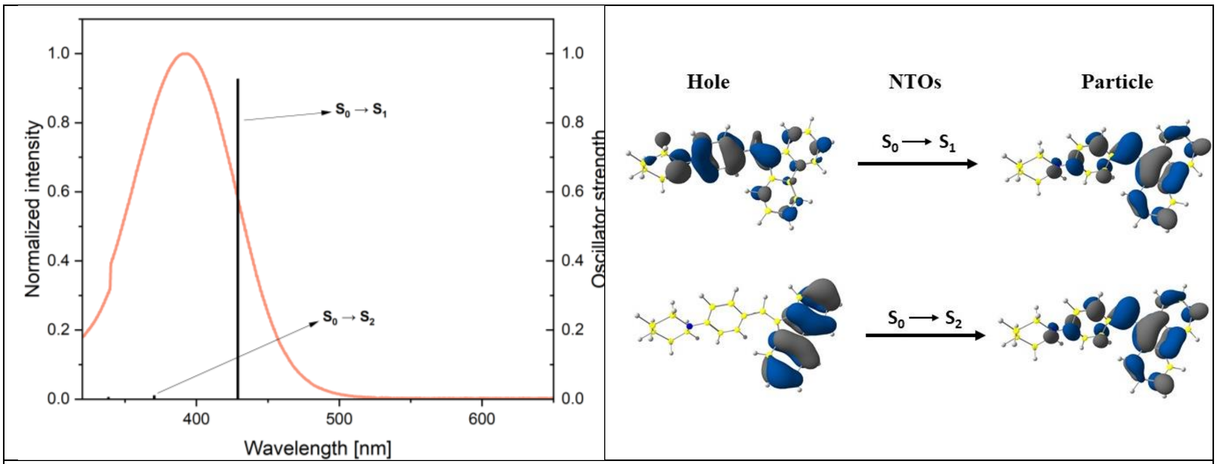

A-4

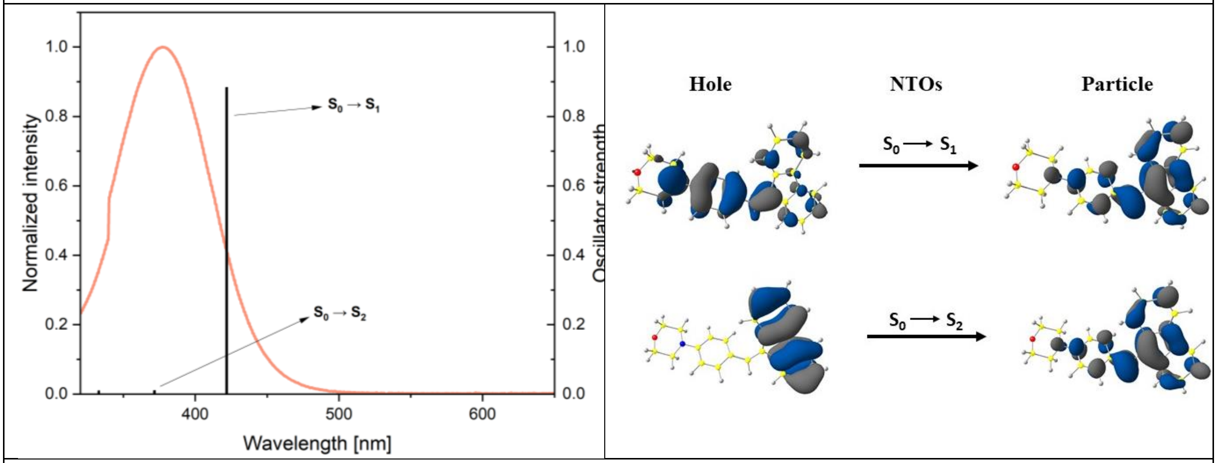

A-5

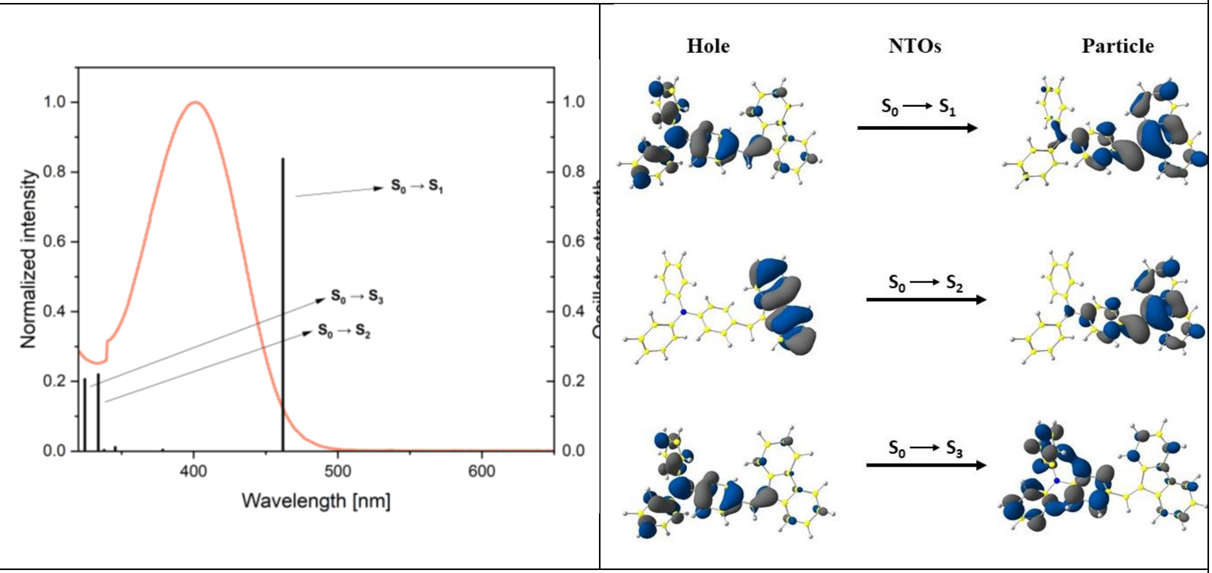

A-6

Figure S21. Electronic spectra of **A-1**–**A-6** in dichloromethane (pink line) alongside national transition orbitals (black sticks) calculated for vertical excitations, which were assigned to the lowest energy absorption band.

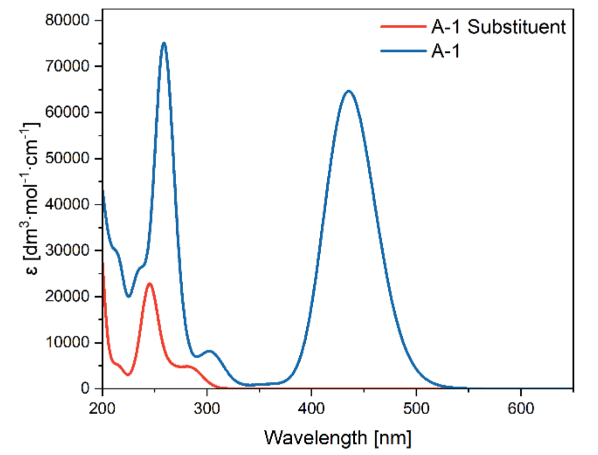

A-1

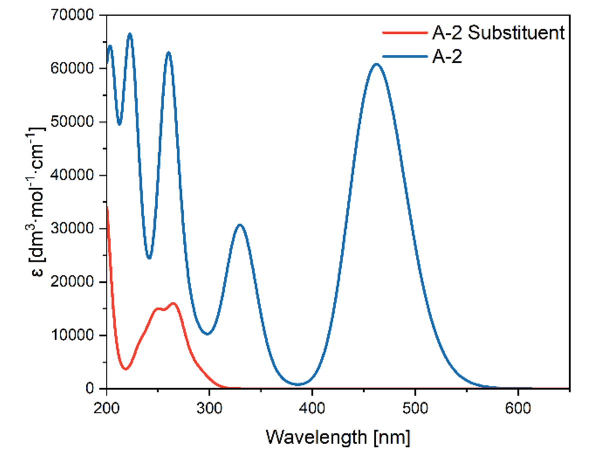

A-2

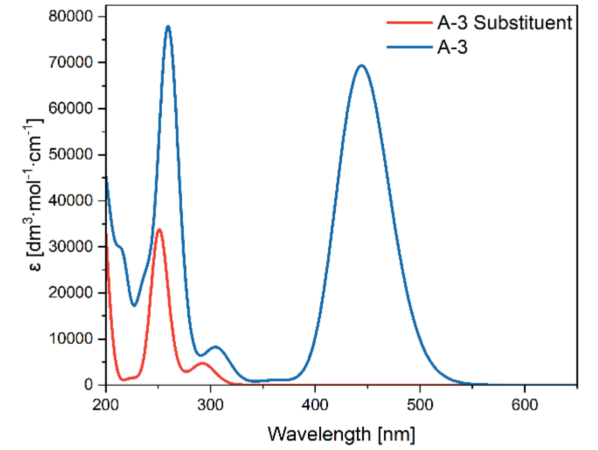

A-3

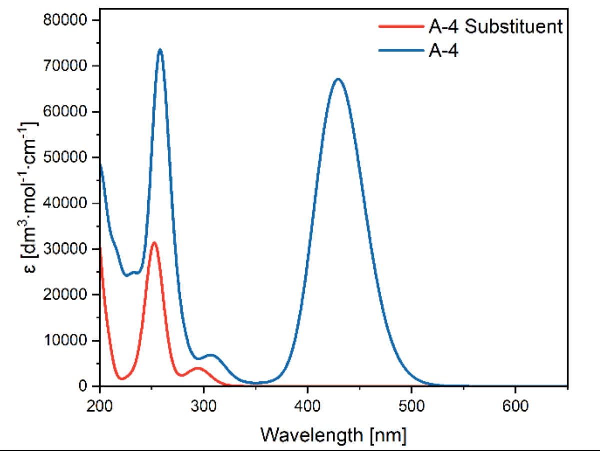

A-4

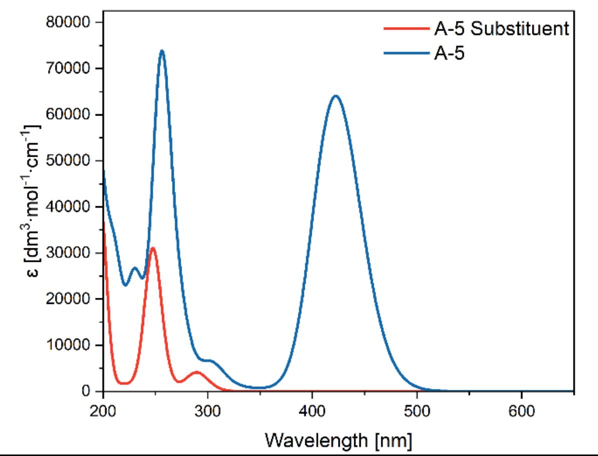

A-5

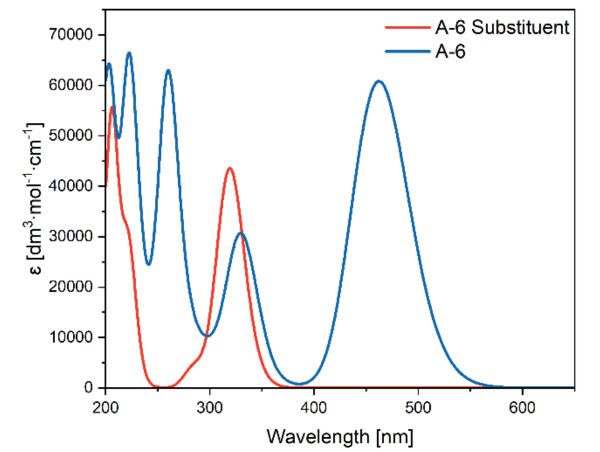

A-6

Figure S22. Comparison of simulated UV–Vis spectra of A-1–A-6 with their N-donor substituents

7. Electrochemistry

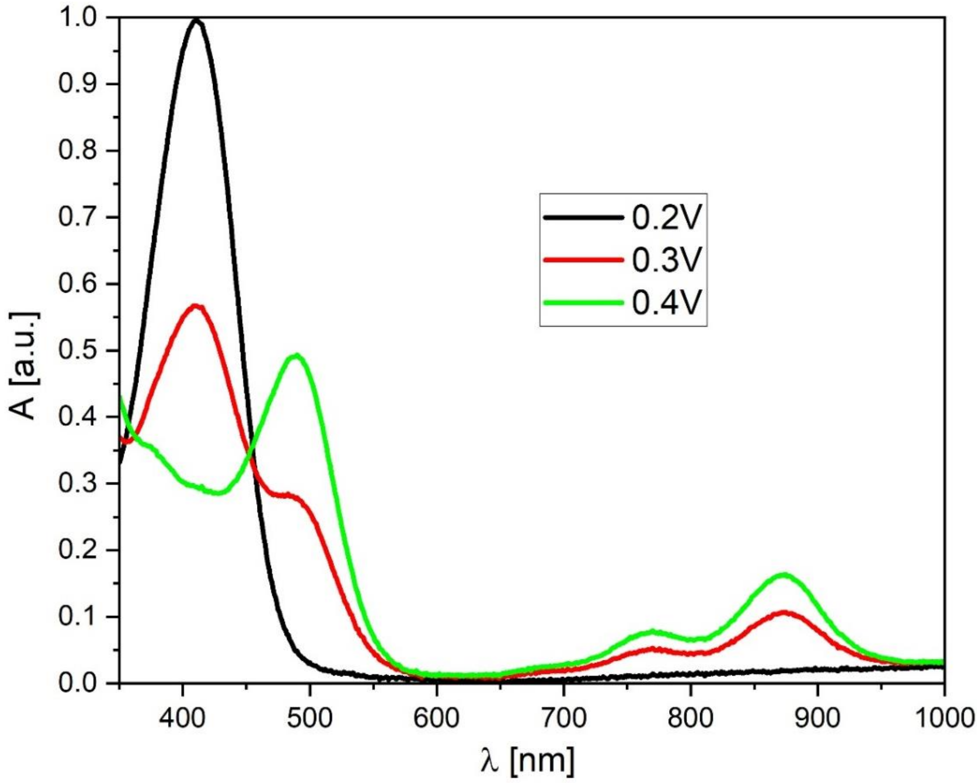

Figure S23. UV–Vis spectro-electrochemistry of the A-3 derivative in MeCN solution (c = 1 × 10^{-5} mol/L, as an inset on each graph, all potentials vs Fc/Fc+ redox couple)

8. Literature

1. Majeed, S.; Junaid, H.M.; Waseem, M.T.; Khan, Z.A.; Khan, A.M.; Shahzad, S.A. Mechanochromic and AIE Active Fluorescent Probes for Solution and Vapor Phase Detection of Picric Acid: Application of Logic Gate. *Journal of Photochemistry and Photobiology A: Chemistry* **2022**, *432*, 114057, doi:10.1016/j.jphotochem.2022.114057.
